# Dual role for Headcase in hemocyte progenitor fate determination in *Drosophila melanogaster*

**DOI:** 10.1371/journal.pgen.1011448

**Published:** 2024-10-28

**Authors:** Bayan Kharrat, Erika Gábor, Nikolett Virág, Rita Sinka, Ferenc Jankovics, Ildikó Kristó, Péter Vilmos, Gábor Csordás, Viktor Honti

**Affiliations:** 1 *Drosophila* Blood Cell Differentiation Group, Institute of Genetics, HUN-REN Biological Research Centre, Szeged, Hungary; 2 Department of Biology, Faculty of Science and Informatics, University of Szeged, Szeged, Hungary; 3 Department of Genetics, Faculty of Science and Informatics, University of Szeged, Szeged, Hungary; 4 Laboratory of *Drosophila* Germ Cell Differentiation, Institute of Genetics, HUN-REN Biological Research Centre, Szeged, Hungary; 5 *Drosophila* Nuclear Actin Laboratory, Institute of Genetics, HUN-REN Biological Research Centre, Szeged, Hungary; 6 Lysosomal Degradation Research Group, Institute of Genetics, HUN-REN Biological Research Centre, Szeged, Hungary; University of Helsinki: Helsingin Yliopisto, FINLAND

## Abstract

The hematopoietic organ of the *Drosophila* larva, the lymph gland, is a simplified representation of mammalian hematopoietic compartments, with the presence of hemocyte progenitors in the medullary zone (MZ), differentiated hemocytes in the cortical zone (CZ), and a hematopoietic niche called the posterior signaling centre (PSC) that orchestrates progenitor differentiation. Our previous work has demonstrated that the imaginal cell factor Headcase (Hdc, Heca) is required in the hematopoietic niche to control the differentiation of hemocyte progenitors. However, the downstream mechanisms of Hdc-mediated hematopoietic control remained unknown. Here we show that Hdc exerts this function by negatively regulating the insulin/mTOR signaling in the niche. When Hdc is depleted in the PSC, the overactivation of this pathway triggers reactive oxygen species (ROS) accumulation and, in turn, the differentiation of effector lamellocytes non-cell-autonomously. Although overactivation of insulin/mTOR signaling normally leads to an increase in the size of the hematopoietic niche, this effect is concealed by cell death caused by *hdc* loss-of-function. Moreover, we describe here that *hdc* silencing in progenitors causes cell-autonomous ROS elevation and JNK pathway activation, resulting in decreased MZ size and differentiation of lamellocytes. Similarly to the PSC niche, knocking down *hdc* in the MZ also leads to caspase activation. Notably, depleting Hdc in the progenitors triggers proliferation, an opposing effect to what is observed in the niche. These findings further our understanding of how progenitor maintenance in the larval lymph gland is controlled autonomously and non-cell-autonomously, and point towards new mechanisms potentially regulating HSC maintenance across vertebrates.

## Author summary

Similarly to stem cell niches, in the hematopoietic organ of the *Drosophila* larva, the lymph gland, the maintenance of stem cells is regulated by both cell-autonomous signals from stem cells themselves, and non-cell-autonomous signals from supporting niche cells. This study describes a dual role for Headcase (Hdc) in progenitor differentiation within the lymph gland. Genetic interactions and rescue experiments revealed that in the niche, Hdc depletion leads to overactivation of the insulin/mTOR pathway, which can exhaust cells over time and lead to the formation of reactive oxygen species (ROS) acting as a non-cell-autonomous signal to induce premature differentiation of progenitors. Interestingly, within the progenitors themselves, ROS elevation due to *hdc* loss-of-function triggers distinct signaling pathways compared to those that function in the niche. We hope that our study contributes to a deeper understanding of regulatory mechanisms involved in progenitor maintenance in *Drosophila*, which would shed light on conserved processes occurring in mammalian stem cell niches.

## Introduction

Hematopoiesis ensures the replenishment of various blood cells through the differentiation of hematopoietic stem cells (HSCs) throughout an individual’s lifetime [1,2]. This crucial process is governed by a complex network of signaling pathways. Any disruptions in this intricate system can lead to the development of blood disorders and cancers [3–5]. Because these signaling pathways are very similar to those involved in blood cell (hemocyte) differentiation in *Drosophila*, the fruit fly became a popular model organism to investigate hematopoiesis [6–8]. In recent decades, numerous immunological and transgenic tools have been developed in *Drosophila* to study blood cell differentiation under naive, immune induced as well as in tumorous conditions [9–13]. Similarly to vertebrates, the hemocytes of *Drosophila* differentiate in multiple waves and are located in distinct hematopoietic compartments in each developmental stage [14]. In the larva, one such compartment is the lymph gland, a multi-lobed organ located alongside the dorsal vessel that stores and supplies hemocytes required for immune response and metamorphosis [15,16]. The lymph gland is generally regarded as a model of HSC niches in mammals [7,8,17]. Based on the expression of cell-type-specific markers, the anterior-most lobes of the lymph gland can be divided into three main zones: the medullary zone (MZ), which contains hemocyte progenitors, the cortical zone (CZ), where differentiated hemocytes reside and the posterior signaling centre (PSC) [17–19]. Two niches, the PSC and the cardiac tube, emit signals to orchestrate the balance between progenitors and differentiated hemocytes in the lymph gland anterior lobes [18–23]. Moreover, progenitors in the medullary zone can be divided based on the varying expression of cellular markers into core progenitors (positive for both MZ markers *Tep4* and *domeMESO*) and distal progenitors (positive for *domeMESO* only) [24,25]. In addition, an intermediate zone (IZ) between the MZ and CZ, consisting of intermediate progenitors expressing both the MZ marker *domeMESO* and the CZ marker *Hml*, was recently described [26–29]. The CZ of the lymph gland contains two distinct types of effector hemocytes that can be also found in the circulation: phagocytic plasmatocytes and crystal cells, a cell type involved in the melanization cascade and wound healing. Following immune induction, a third type of blood cells, the lamellocytes, appear in the circulation and the lymph gland. Lamellocytes play an indispensable role in the encapsulation of invaders, such as the egg of the parasitic wasp *Leptopilina boulardi* [6,30–32]. Since these cells are not present under uninduced conditions, their appearance in naive animals can indicate a dysfunction in hemocyte progenitor maintenance mechanisms in the larva [33,34], similarly to what was observed earlier in *headcase* (*hdc*, *heca*) mutants.

Hdc, the orthologue of the human tumor suppressor HECA, is a cytoplasmic protein that is present in larval stem cell-like and organ precursor cells [35]. Up to now, Hdc has been linked to various developmental processes, such as imaginal disc morphogenesis, maintenance of the testis niche cells and intestinal stem cells, and the regulation of tracheal branching [35–41]. In the hematopoietic system, *hdc* is expressed exclusively in the lymph gland, and its loss-of-function in the PSC leads to lamellocyte differentiation [35,42,43]. This phenotype can be rescued by the overexpression of Hedgehog (Hh) and Decapentaplegic (Dpp), two signaling molecules that play a crucial role in progenitor maintenance, suggesting that Hdc functions upstream to these pathways in the niche. Interestingly, Hdc depletion in the niche does not affect PSC size nor the identity of niche cells [43]. The involvement of Hdc in the regulation of imaginal cell fate via the insulin/mTOR pathway raised the question whether its phenotype in the hematopoietic niche is mediated through the same mechanism [39–41].

Here we show that in the PSC niche, *hdc* depletion activates the insulin/mTOR pathway and leads to the subsequent elevation of ROS levels, which triggers non-cell-autonomous lamellocyte differentiation in the lymph gland. Moreover, we reveal that in these larvae, the expected increase in the PSC size due to the elevated activity of the insulin/mTOR pathway is compensated by cell death [44,45]. In addition to its role in the niche, we also unveil that Hdc cell-autonomously controls hemocyte progenitor fate in the MZ. Although this role is also dependent on ROS levels, it is conveyed through Jun N-terminal kinase (JNK) pathway and other signaling mechanisms distinct from those involved in the PSC niche.

## Results

### Hdc negatively regulates the insulin/mTOR pathway in the PSC

**Fig 1:**
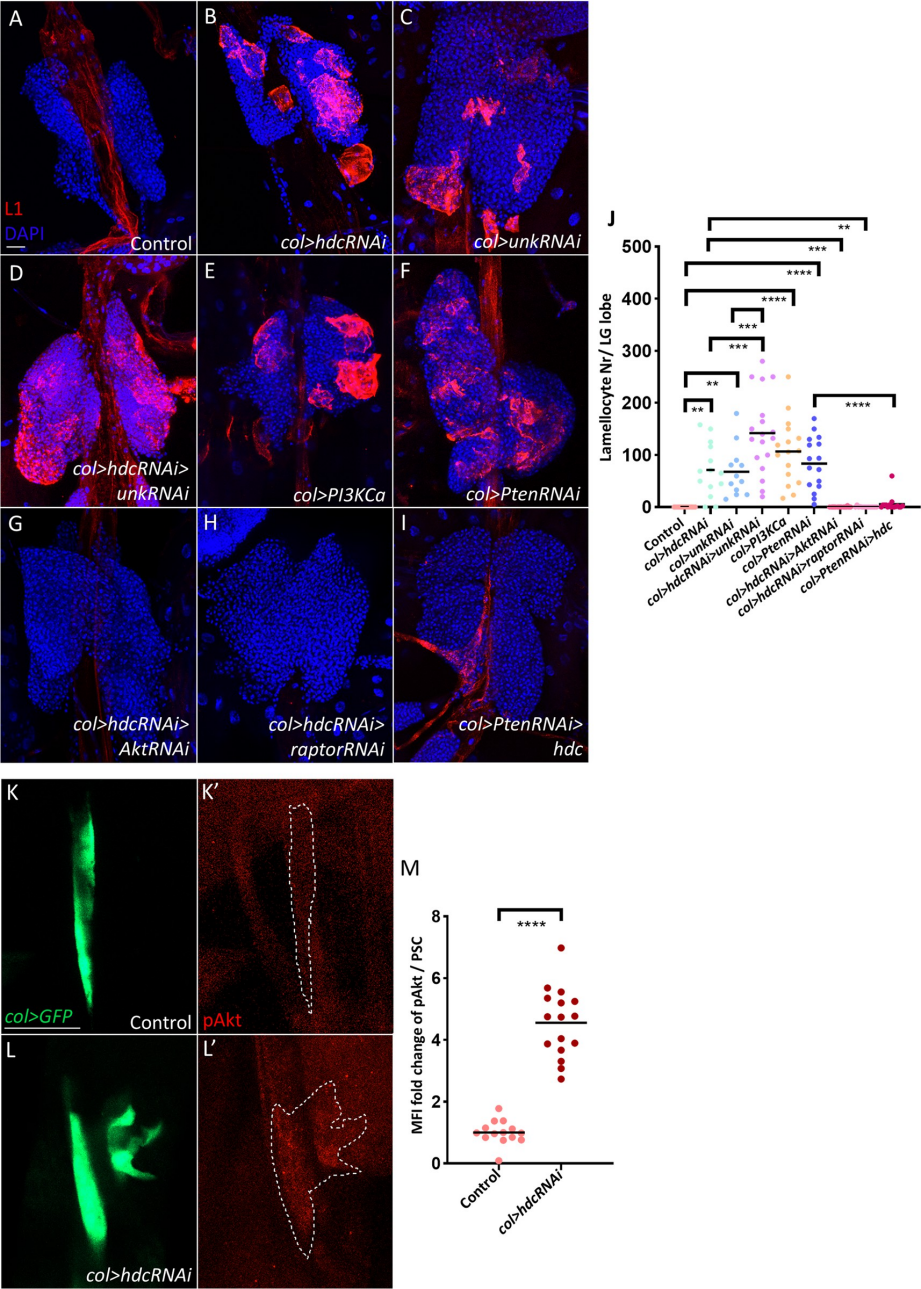
Hdc negatively regulates the insulin/mTOR pathway in the PSC. (A-F) Lamellocytes (red) are absent from control lymph glands (*Pcol85-Gal4/+*) (n = 14) (A), while they are present when *hdc* is silenced in the PSC (*Pcol85-Gal4*,*UAS-hdcRNAi/+*) (n = 14) (B), its partner *unk* is silenced (*Pcol85-Gal4/UAS-unkRNAi)* (n = 12) (C), *hdc* and *unk* are silenced together (*Pcol85-Gal4*,*UAS-hdcRNAi/UAS-unkRNAi*) (n = 18) (D), a constitutively active *Pi3K* is expressed (*UAS-Pi3K92E*.*CAAX/+; Pcol85-Gal4/+*) (n = 16) (E), or the negative insulin pathway regulator *Pten* is silenced (*Pcol85-Gal4/+; UAS-PtenRNAi/+*) (n = 16) (F). (G-H) Lamellocyte differentiation in lymph glands of *col>hdcRNAi* larvae is rescued when simultaneously either *Akt* (*Pcol85-Gal4*,*UAS-hdcRNAi/+; UAS-AktRNAi/+*) (n = 22) (G) or *raptor* (*Pcol85-Gal4*,*UAS-hdcRNAi/+; UAS-raptorRNAi/+*) (n = 14) is silenced (H). (I) Overexpression of *hdc* rescues lamellocyte differentiation in *col>PtenRNAi* larvae (*Pcol85-Gal4/+; UAS-PtenRNAi/UAS-hdc*.*S*) (n = 16). *n* refers to the number of lymph gland lobes analyzed. Nuclei are visualized by DAPI (blue). Scale bar: 20 μm. (J) Scatter plot of the number of lamellocytes per lymph gland lobe in the genotypes presented in panels (A-I). Each dot in the graph represents one lymph gland lobe. Data were analyzed using ANOVA with Tukey’s test for multiple comparisons, ** *p* ≤ 0.01, *** *p* ≤ 0.001, **** *p* ≤ 0.0001. (K-L’) pAkt antibody staining (red) detects higher levels of pAkt in the niche (*col>GFP* positive cells, green) when *hdc* is silenced (*Pcol85-Gal4*,*UAS-2xEGFP/UAS-hdcRNAi*) (n = 16) (L-L’) in comparison to control (*Pcol85-Gal4*,*UAS-GFP/+*) (n = 14) (K-K’). *n* refers to the number of lymph gland lobes analyzed. Scale bar: 20 μm. (M) A scatter dot plot showing the fold change (average = 4.5 folds) increase in the mean fluorescence intensity (MFI) of pAkt in the PSC (*col>GFP* positive cells) of *col>hdcRNAi* (*Pcol85-Gal4*,*UAS-2xEGFP/UAS-hdcRNAi*) larvae in comparison to the control (*Pcol85-Gal4*,*UAS-2xEGFP/+*). Each dot in the graph represents a PSC from one lobe. Data were analyzed using two-tailed unpaired Student’s t-test, **** *p* ≤ 0.0001.

In a previous study, we showed that silencing *hdc* in the posterior signaling centre (referred to as PSC, or niche from hereon) results in lamellocyte differentiation without immune induction (Figs 1A, 1B, S1A and S1B, and quantified in 1J and S1J) [43]. To better understand the downstream mechanisms of how Hdc controls hematopoiesis, we carried out genetic interaction experiments in the PSC. We found that silencing *unk*, a gene encoding a physical interacting partner of Hdc [39,40], resulted in a similar phenotype (Figs 1C and S1C, quantified in 1J and S1J), while silencing *unk* together with *hdc* significantly increased lamellocyte numbers compared to *col>hdcRNAi* animals (Figs 1D and S1D, quantified in 1J and S1J). Since both Hdc and Unk were previously described to bind to the mTOR component Raptor and inhibit mTOR activity in the imaginal discs [40], we set out to explore whether the insulin/mTOR pathway is involved in the *hdc* loss-of-function phenotype in the PSC. To confirm whether activating the insulin/mTOR pathway in the niche mimics the *hdc*-depleted phenotype, we overexpressed the constitutively active form of the *Pi3K* kinase (*Pi3KCa*), and silenced the negative regulator *Pten*, both of which lead to the appearance of lamellocytes in the lymph gland and in the circulation (Figs 1E, 1F, S1E and S1F, and quantified in 1J and S1J), as previously described [45,46]. To examine if the insulin pathway is activated in the niche of *col>hdcRNAi* larvae, we analyzed the levels of phosphorylated Akt (pAkt), the activated form of the main kinase of insulin/mTOR signaling [47], and found that it was significantly upregulated in the niche upon *hdc* silencing compared to the control (Fig 1K–1L’, quantified in 1M). In line with this, silencing Akt or Raptor in *col>hdcRNAi* larvae counteracted lamellocyte differentiation in the lymph gland and their appearance in the circulation (Figs 1G, 1H, S1G and S1H and quantified in 1J and S1J). This result confirms that the activity of the insulin/mTOR pathway is responsible for lamellocyte differentiation when Hdc is depleted in the niche. Interestingly, overexpression of *hdc* significantly inhibited lamellocyte differentiation when the insulin/mTOR pathway was overactivated in the PSC (*col>PtenRNAi*, Figs 1I and S1I, quantified in 1J and S1J). Together, these results suggest that Hdc is a suppressor of the insulin/mTOR pathway in the PSC, and depletion of Hdc in the niche (*col>hdcRNAi*) leads to continuous insulin/mTOR activation and induces lamellocyte differentiation in the lymph gland.

### Hdc depletion leads to apoptosis and cell cycle arrest in the PSC independently from the insulin/mTOR pathway

**Fig 2:**
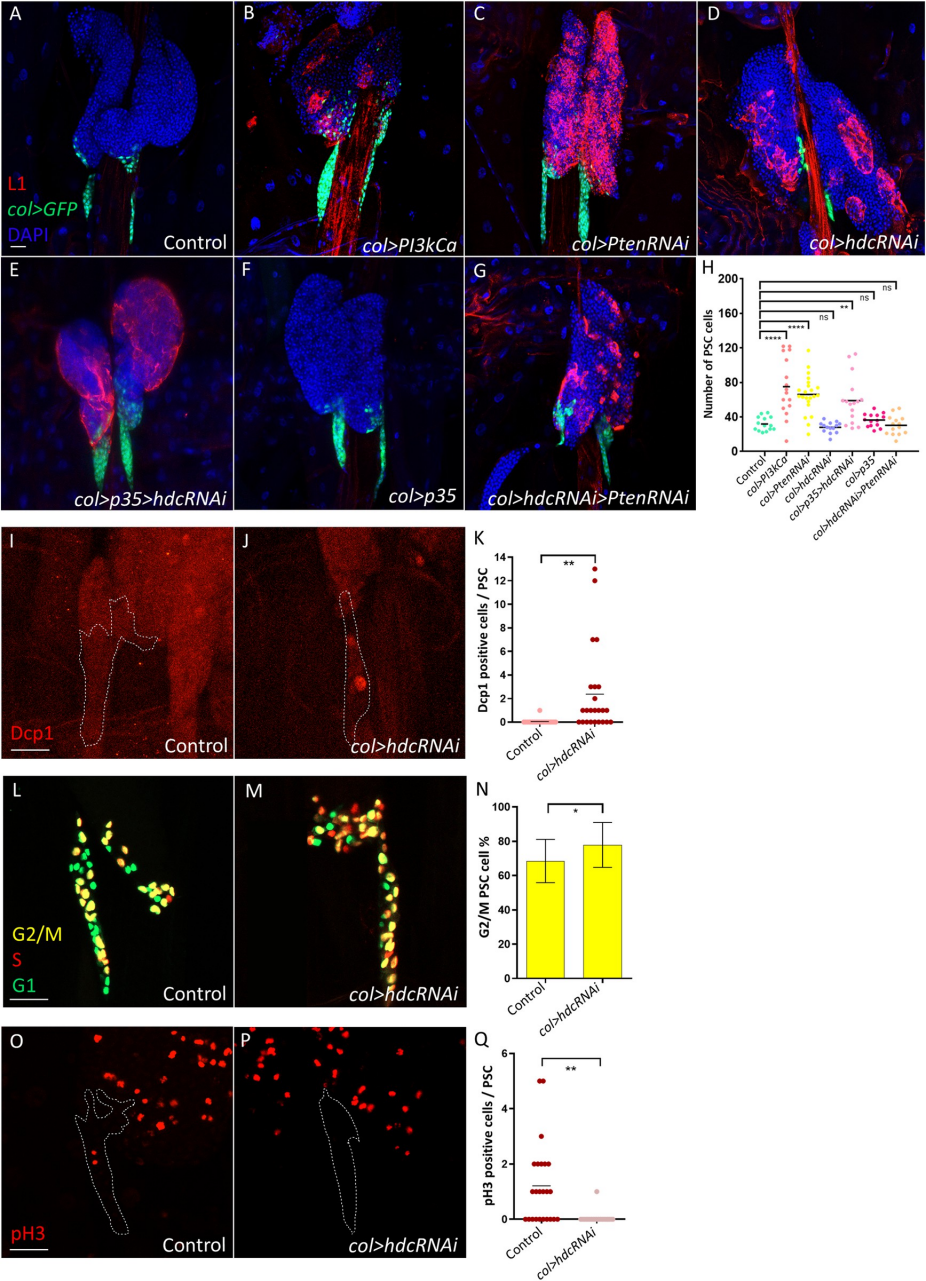
*hdc* silencing causes cell death and cell cycle arrest in the PSC. (A-C) An enlargement of the PSC (number of *col>GFP* positive cells) is observed when the insulin/mTOR pathway is activated by expressing *Pi3KCa* (*UAS-Pi3K92E*.*CAAX; Pcol85-Gal4*,*UAS-2xEGFP/+*) (average number of PSC cells = 75, n = 16) (B) or silencing *Pten* (*Pcol85-Gal4*,*UAS-2xEGFP/+;UAS-PtenRNAi*) (average number of PSC cells = 66, n = 22) (C) in comparison to the control (*Pcol85-Gal4*,*UAS-2xEGFP/+*) (average number of PSC cells = 31, n = 14) (A). (D-F) Silencing *hdc* alone does not alter PSC size (*Pcol85-Gal4*,*hdcRNAi/+; UAS-2xEGFP/+*) (average number of PSC cells = 28, n = 14) (D), while simultaneous overexpression of the apoptosis inhibitor *p35* increases PSC cell numbers (*Pcol85-Gal4*,*hdcRNAi/+; UAS-2xEGFP/UAS-p35*) (average number of PSC cells = 59, n = 16) (E), a phenotype not observed in case *p35* is overexpressed alone (*Pcol85-Gal4*,*UAS-2xEGFP/+; UAS-p35/+)* (average number of PSC cells = 36, n = 14) (F). (G) Silencing *hdc* simultaneously with *Pten* reduces PSC size of *col>PtenRNAi* larvae to normal (*Pcol85-Gal4*,*hdcRNAi/+; UAS-2xEGFP/UAS-PtenRNAi*) (average number of PSC cells = 30, n = 14) (blue: nuclei, green: PSC, red: lamellocytes). *n* refers to the number of lymph gland lobes analyzed. Scale bar: 20 μm. (H) A scatter dot plot showing PSC cell number in larvae from the genotypes presented in panels (A-G). Each dot in the graph represents a PSC from one lymph gland lobe. Data were analyzed using ANOVA with Tukey’s test for multiple comparisons, ** *p* ≤ 0.01, **** *p* ≤ 0.0001, ns: non-significant. (I-J) Apoptotic cells (Dcp1 positive, red) can be observed at a higher frequency when *hdc* is silenced in the niche (*Pcol85-Gal4*,*UAS-2xEGFP/UAS-hdcRNAi*) (n = 16) (J) in comparison to the control (*Pcol85-Gal4*,*UAS-2xEGFP/+*) (n = 14) (I). *n* refers to the number of lymph gland lobes analyzed. Scale bar: 20 μm. (K) A scatter dot plot quantifying the number of Dcp1 positive cells in the niches (*col>GFP* positive cells) of genotypes presented in the panels (I-J). Each dot in the graph represents a PSC from one lymph gland lobe. Data were analyzed using two-tailed unpaired Student’s t-test, ** *p* ≤ 0.01. (L-M) FUCCI cell cycle reporter pattern (green: G1 phase, red: S phase, yellow: G2/M phase) in the niche of *col>hdcRNAi* (*Pcol85-Gal4*,*UAS-hdcRNAi/+; UAS-EGFP*::*E2F1*^*1-230*,^*UAS-mRFP1*::*CycB*^*1-266*^*)* (n = 20) (M) in comparison to the control (*Pcol85-Gal4/+; UAS-EGFP*::*E2F1*^*1-230*,^*UAS-mRFP1*::*CycB*^*1-266*^) (n = 22) (L). *n* refers to the number of lymph gland lobes analyzed. Scale bar: 20 μm. (N) A bar graph showing the mean and standard deviation of the number of niche cells in the G2/M phase in the genotypes presented in the panels (L-M). Data were analyzed using two-tailed unpaired Student’s t-test, * *p* ≤ 0.05. (O-P) Dividing cells (pH3 positive, red) can be observed at a lower frequency when *hdc* is silenced in the niche (*Pcol85-Gal4*,*UAS-2xEGFP /UAS-hdcRNAi*) (n = 20) (P) in comparison to the control (*Pcol85-Gal4*,*UAS-2xEGFP/+*) (n = 24) (O). *n* refers to the number of lymph gland lobes analyzed. Scale bar: 20 μm. (Q) A scatter dot plot quantifying the number of pH3 positive cells in the niches (*col>GFP* positive cells) of genotypes presented in the panels (O-P). Each dot in the graph represents a PSC from one lymph gland lobe. Data were analyzed using two-tailed unpaired Student’s t-test, ** *p* ≤ 0.01.

Previously, it was shown that continuous activation of the insulin/mTOR pathway leads to an increase in PSC size [44–46]. Indeed, we found that both *Pi3KCa* overexpression and *Pten* silencing resulted in significantly higher PSC cell numbers as compared to the control (Fig 2A–2C, quantified in 2H). However, in case of *hdc* silencing, the number of PSC cells remained comparable to that of the control (Fig 2D, quantified in 2H), which underlines the original observation by Varga et al. (2019) [43]. One potential interpretation of this phenomenon is that cell death caused by *hdc* silencing conceals the effect of insulin/mTOR. To examine this possibility, we simultaneously overexpressed the apoptosis inhibitor *p35* while silencing *hdc* in the niche. Interestingly, we found that this led to a significant increase of PSC cell number, which was not observed with *p35* overexpression alone (Fig 2E and 2F, quantified in 2H). Moreover, we found that silencing *hdc* restores the number of PSC cells in *col>PtenRNAi* larvae to normal (Fig 2G, quantified in 2H), further confirming that Hdc depletion causes cell death, which compensates for the expansion in PSC cell number in case of *hdc* loss-of-function. Although we observed significantly more apoptotic PSC cells in *col>hdcRNAi* niches in comparison to the control as assayed by immunostaining of the apoptosis marker cleaved Dcp1 (*Drosophila* caspase-1) [48] (Fig 2I, 2J, quantified in 2K), PSC cell number appeared normal in third instar larvae, which suggests that cell death is likely triggered in earlier developmental stages. Moreover, analysis of the cell cycle profile of PSC cells in *col>hdcRNAi* larvae with the cell cycle reporter FUCCI [49] revealed that, similarly to what was observed recently in MZ progenitors [50], most PSC cells are normally paused in G2/M phase, with a significant increase when *hdc* is silenced in the PSC (Fig 2L, 2M, quantified in 2N). To determine whether these cells are arrested in the G2 phase or dividing in the M phase, we immunostained the phospho-Histone H3 (pH3) mitosis marker. We found significantly fewer dividing PSC cells when *hdc* was silenced (Fig 2O, 2P, quantified in 2Q), which suggests that Hdc depletion not only causes apoptosis in the niche but also leads to cell cycle arrest.

### *hdc* silencing in the niche leads to ROS production, which triggers lamellocyte differentiation

**Fig 3:**
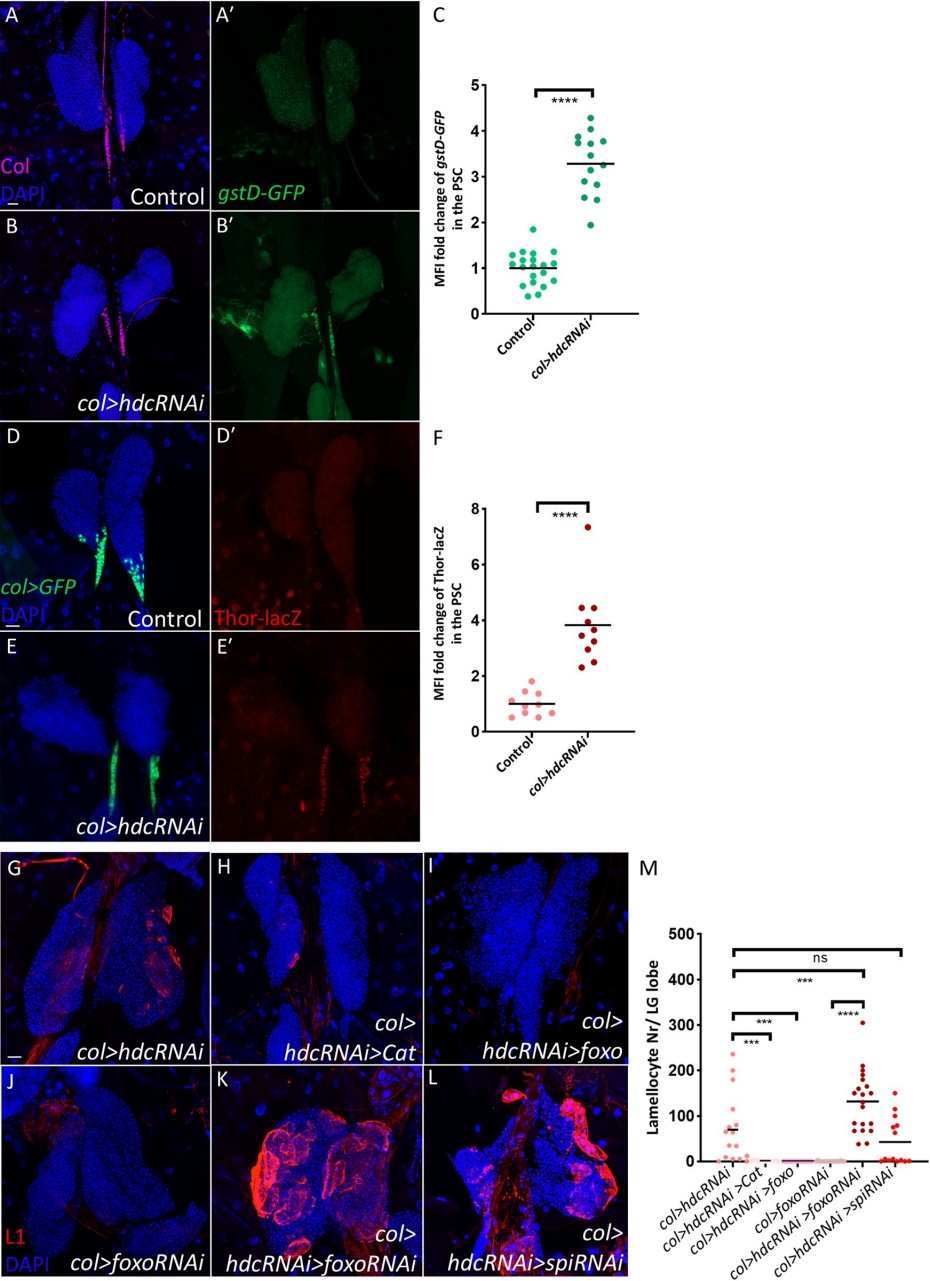
*hdc* silencing results in cellular stress in the hematopoietic niche. (A-B’) Silencing *hdc* results in the activation of the *gstD-GFP* reporter in the PSC (Col antibody positive area) (*Pcol85-Gal4*,*UAS-hdcRNAi/gstD-GFP*) (n = 14) (B-B’), in comparison to the control (*Pcol85-Gal4/gstD-GFP*) (n = 20) (A-A’) (blue: nuclei, green: ROS, magenta: Collier). *n* refers to the number of lymph gland lobes analyzed. Scale bar: 20 μm. (C) A scatter dot plot showing the fold change (average = 3.2 folds) increase in the mean fluorescence intensity (MFI) of *gstD-GFP* in the PSC of *col>hdcRNAi* (*Pcol85-Gal4*,*UAS-hdcRNAi/gstD-GFP*) larvae in comparison to the control (*Pcol85-Gal4/gstD-GFP*). Each dot in the graph represents a PSC from one lobe. Data were analyzed using two-tailed unpaired Student’s t-test, **** *p* ≤ 0.0001. (D-E’) Silencing *hdc* induces the transcription of *Thor* in the PSC (*col>GFP* positive area) as detected by an anti-lacZ staining for the *Thor-lacZ* reporter (*Pcol85-Gal4*,*UAS-hdcRNAi/Thor-lacZ*) (n = 10) (E-E’) in comparison to the control (*Pcol85-Gal4/Thor-lacZ*) (n = 10) (D-D’) (blue: nuclei, green: PSC, red: Thor-LacZ). *n* refers to the number of lymph gland lobes analyzed. Scale bar: 20 μm. (F) A scatter dot plot showing the fold change (average = 3.8 folds) increase in MFI of Thor-LacZ in the PSC cells of *col>hdcRNAi* (*Pcol85-Gal4*,*UAS-hdcRNAi/Thor-lacZ*) larvae compared to the control (*Pcol85-Gal4/Thor-lacZ*). Each dot in the graph represents a PSC from one lobe. Data were analyzed using two-tailed unpaired Student’s t-test, **** *p* ≤ 0.0001. (G-I) Overexpression of *Cat* (*Pcol85-Gal4*,*UAS-hdcRNAi/UAS-Cat*) (n = 18) (H) and *foxo* (*Pcol85-Gal4*,*UAS-hdcRNAi/UAS-foxo*) (n = 20) (I) rescues lamellocyte differentiation in the lymph glands of *col>hdcRNAi* (*Pcol85-Gal4*,*UAS-hdcRNAi/+*) larvae (n = 14) (G). (J-K) Silencing *foxo* (*Pcol85-Gal4*,*UAS-hdcRNAi/+; UAS-foxoRNAi/+*) (n = 20) enhances lamellocyte differentiation in *col>hdcRNAi* lymph glands (K), while silencing *foxo* alone (*Pcol85-Gal4/+; UAS-foxoRNAi/+*) does not lead to lamellocyte differentiation (n = 18) (J). (L) Silencing *spi* does not affect lamellocyte differentiation in *col>hdcRNAi* lymph glands (*Pcol85-Gal4*,*UAS-hdcRNAi/UAS-spiRNAi*) (n = 14) (blue: nuclei, red: lamellocytes). *n* refers to the number of lymph gland lobes analyzed. Scale bar: 20 μm. (M) A scatter dot plot showing the number of lamellocytes per lymph gland lobe in the genotypes presented in panels (G-L). Each dot in the graph represents one lymph gland lobe. Data were analyzed using ANOVA with Tukey’s test for multiple comparisons, *** *p* ≤ 0.001, **** *p* ≤ 0.0001, ns: non-significant.

In the hematopoietic niche, continuous activation of the insulin/mTOR pathway was demonstrated to result in ROS accumulation [45]. Elevated ROS levels serve as a non-cell autonomous signal that induces progenitor differentiation in the lymph gland [15,45,51]. To investigate whether silencing *hdc* leads to higher ROS levels in the PSC, we used *gstD-GFP*, a transgenic reporter, in which the oxidative stress-sensitive enhancer of *glutathione S transferase D1* gene drives the expression of GFP [52]. Consistent with our expectation, a clear induction of the *gstD-GFP* reporter was observed in the niche of *col>hdcRNAi* larvae as compared to controls (Fig 3A–3B’, quantified in 3C). The same was observed using the ROS sensitive dye dihydroethidium (DHE) (S2A–S2B Fig, quantified in S2D) [53,54]. Furthermore, the Forkhead box O (Foxo) reporter (*Thor-lacZ*), which was shown previously to become active upon ROS production in the niche [51,55–57], was upregulated in response to *hdc* silencing (Fig 3D–3E’, quantified in 3F), further confirming that Hdc depletion in the niche causes cellular stress. To investigate if insulin/mTOR pathway activation in the *col>hdcRNAi* niche is the reason behind ROS accumulation, we simultaneously silenced *Akt* and *hdc*, and found that this significantly rescued the higher ROS levels observed in the *col>hdcRNAi* niche (S2A–S2C Fig, quantified in S2D). This further supports the notion that the continuous activation of the insulin/mTOR pathway, in the absence of Hdc, is the driving force behind ROS accumulation.

Next, we asked whether ROS accumulation is the reason behind niche cell apoptosis or lamellocyte differentiation upon PSC-specific *hdc* knockdown. To investigate this, we overexpressed Catalase, an enzyme responsible for breaking down ROS, and FoxO, a transcription factor that stimulates the expression of antioxidant enzymes ​[58,59].

Interestingly, we found that neither *Catalase*, nor *foxo* overexpression impacted PSC size in *col>hdcRNAi* larvae (S2E–S2G Fig, and quantified in S2H), suggesting that apoptosis in the PSC is not triggered by elevated ROS levels. However, in both cases we observed the rescue of lamellocyte differentiation (Figs 3G–3I, S3A–S3C, quantified in 3M and S3G), while silencing *hdc* and *foxo* together enhanced lamellocyte differentiation (Figs 3J, 3K, S3D, S3E and quantified in 3M and S3G). These results indicate that the niche-specific accumulation of ROS is indeed the catalyst for lamellocyte differentiation upon *hdc* loss.

Notably, literature data has suggested that lamellocyte differentiation in response to oxidative stress in the niche is mediated through the secretion of the EGFR ligand Spitz (Spi) from the PSC [45,51]. However, we found that knocking down *spi* in *col>hdcRNAi* larvae did not abolish lamellocyte differentiation (Figs 3L and S3F, quantified in 3M and S3G), suggesting the involvement of either another EGFR ligand or another non-cell-autonomous mechanism. Taken together, our results suggest that the induction of the insulin/mTOR pathway in *col>hdcRNAi* larvae leads to cellular stress and ROS overproduction, which consequently prompts lamellocyte differentiation in a non-cell-autonomous manner.

### Hdc functions cell-autonomously in the MZ to suppress lamellocyte differentiation

**Fig 4:**
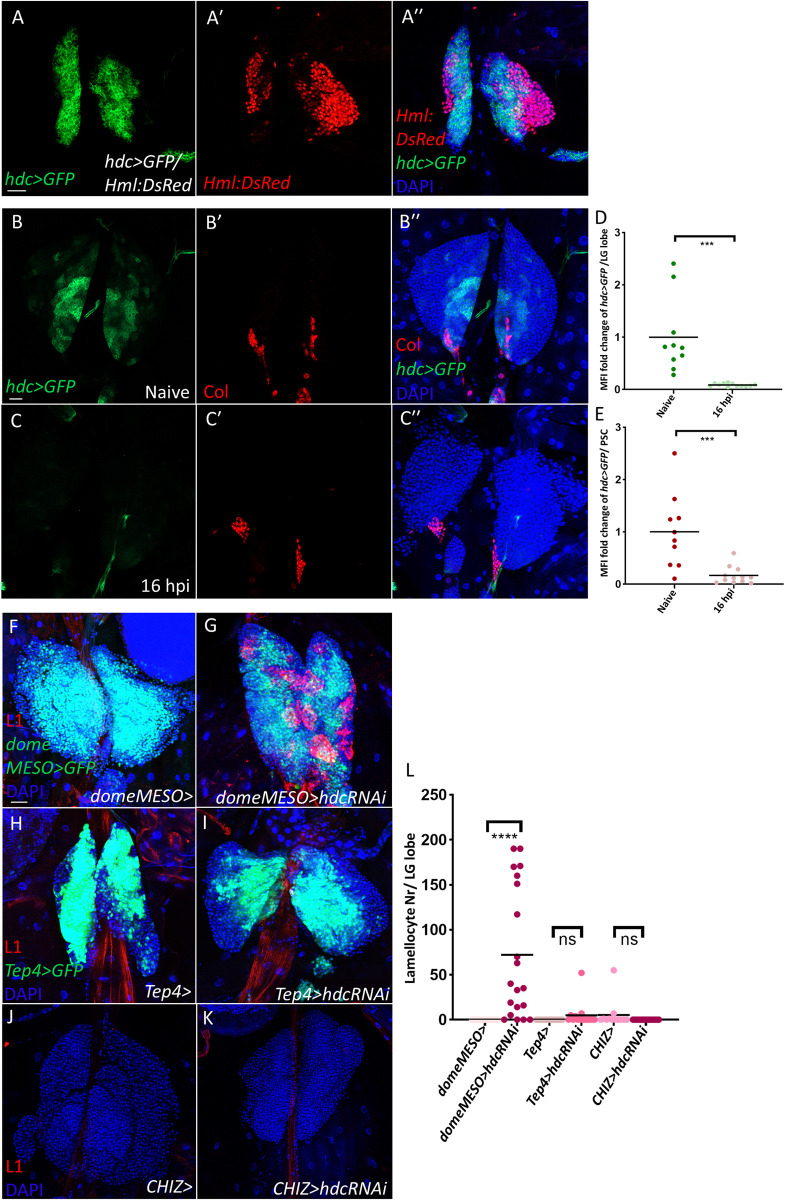
A cell-autonomous role of Hdc in the medullary zone. (A-A”) *hdc>GFP* shows complementary expression to the CZ marker *Hml*:*DsRed* (*UAS-mCD8*::*GFP; hdc*^*19*^*-Gal4/Hml*:*DsRed)* (n = 16) (blue: nuclei, green: *hdc*, red: CZ). Scale bar: 20 μm. (B-C”) *hdc* expression decreases drastically in the lymph gland 16 hpi (n = 14) (C-C”) in comparison to naive control lymph glands (*UAS-mCD8*::*GFP/+; hdc*^*19*^*-Gal4/+)* (n = 10) (B-B”) (blue: nuclei, green: *hdc*, red: Col). Scale bar: 20 μm. (D-E) A scatter dot plot showing the fold change decrease in MFI of *hdc>GFP* in per lymph gland lobe (average = -12.5 folds) (D) and per PSC (Col antibody positive area) (average = -6.25 folds) (E) of wasp infested (16 hpi) larvae compared to the control (*UAS-mCD8*::*GFP/+; hdc*^*19*^*-Gal4/+)*. Each dot in the graph represents one anterior lobe. Data were analyzed using two-tailed unpaired Student’s t-test, *** *p* ≤ 0.001. (F-G) Silencing *hdc* in the MZ of the lymph gland leads to lamellocyte differentiation (*UAS-hdcRNAi/+; domeMESO-GAL4*,*UAS-2xEGFP/+*) (n = 20) (G), while lamellocytes are normally not detected in the control (*domeMESO-GAL4*,*UAS-2xEGFP/+*) (n = 20) (F) (blue: nuclei, green: MZ, red: lamellocytes). (H-I) Similarly to the control (*Tep4-Gal4*; *UAS-2xEGFP/+*) (n = 14) (H), lamellocytes are not observed when *hdc* is silenced using the *Tep4-Gal4* specific for core progenitors (*Tep4-Gal4*; *UAS-hdcRNAi/+; UAS-2xEGFP/+*) (n = 14) (I) (blue: nuclei, green: core progenitors, red: lamellocytes). (J-K) Like in the control (*CHIZ-Gal4*/*+*) (n = 12) (J), lamellocytes do not differentiate in the lymph gland when *hdc* is silenced using the *CHIZ-Gal4* specific for the IZ (*CHIZ-Gal4*/*UAS-hdcRNAi*) (n = 16) (K) (blue: nuclei, red: lamellocytes). *n* refers to the number of lymph gland lobes analyzed. Scale bar: 20 μm. (L) A scatter dot plot showing the number of lamellocytes per lymph gland lobe in the genotypes presented in panels (F-K). Each dot in the graph represents one lymph gland lobe. Data were analyzed using ANOVA with Tukey’s test for multiple comparisons, **** *p* ≤ 0.0001, ns: non-significant.

Previously, we have demonstrated that *hdc* is expressed abundantly in the anterior lymph gland lobes of second instar larvae, and its expression gradually diminishes with the formation and expansion of the CZ from the beginning of the third instar larval stage [43]. This is visible in Fig 4A–4A”, where *hdc* shows an expression pattern complementary to the CZ marker *Hml*:*DsRed*. By the end of the third instar larval stage, *hdc* expression disappears entirely from the anterior lobes as most progenitors undergo differentiation and the lymph gland starts to disintegrate to release its content into the circulation [43]. This changing expression pattern of *hdc* during development intrigued us to investigate how its expression would be altered by immune induction with the parasitic wasp *Leptopilina boulardi*. To capture the moment that precedes the disintegration of the anterior lobes, which is estimated to occur 20 hours after wasp infestation [15], we examined lymph glands 16 hours post infestation (hpi). We found that, similarly to what was observed at the end of the larval stage, *hdc* expression was no longer detectable in the anterior lobes or in the PSC (Fig 4B–4C”, quantified in 4D and 4E). This temporal expression pattern of *hdc* during development and immune response suggests that, in addition to its non-cell-autonomous regulatory function in the PSC, Hdc might also be cell-autonomously involved in preserving progenitor cells in an undifferentiated state. To investigate this possibility, we silenced *hdc* selectively in the MZ using the *domeMESO-Gal4* driver [60]. Consequently, we observed that lamellocytes differentiated in the lymph gland and appeared in the circulation (Figs 4F, 4G, S4A and S4B and quantified in 4L and S4H). When *hdc* was silenced using the core progenitor-specific driver *Tep4-Gal4* or with the IZ-specific driver *CHIZ-Gal4* [25,27,34], lamellocyte differentiation was not triggered (Fig 4H–4K, quantified in 4L), suggesting that Hdc is responsible for suppressing this cell fate in distal progenitors. Moreover, although we did not see a difference in the amount of P1 positive cells in *domeMESO>hdcRNAi* lymph glands (S5A and S5B Fig, quantified in S5D), we found a significant decrease in the crystal cell index compared to the control (S5E and S5F Fig, quantified in S5H). The same phenomenon was observed when *hdc* was silenced in the niche (S5I, S5J, S5L, and S5M Fig, quantified in S5K and S5N), which, in agreement with previous results, suggests that lamellocytes differentiate in the lymph gland at the expense of crystal cells [29].

Taken together, these results reveal an additional cell-autonomous role for Hdc in the MZ progenitors to suppress their premature differentiation into lamellocytes.

### *hdc* silencing in the MZ causes ROS-induced lamellocyte differentiation

**Fig 5:**
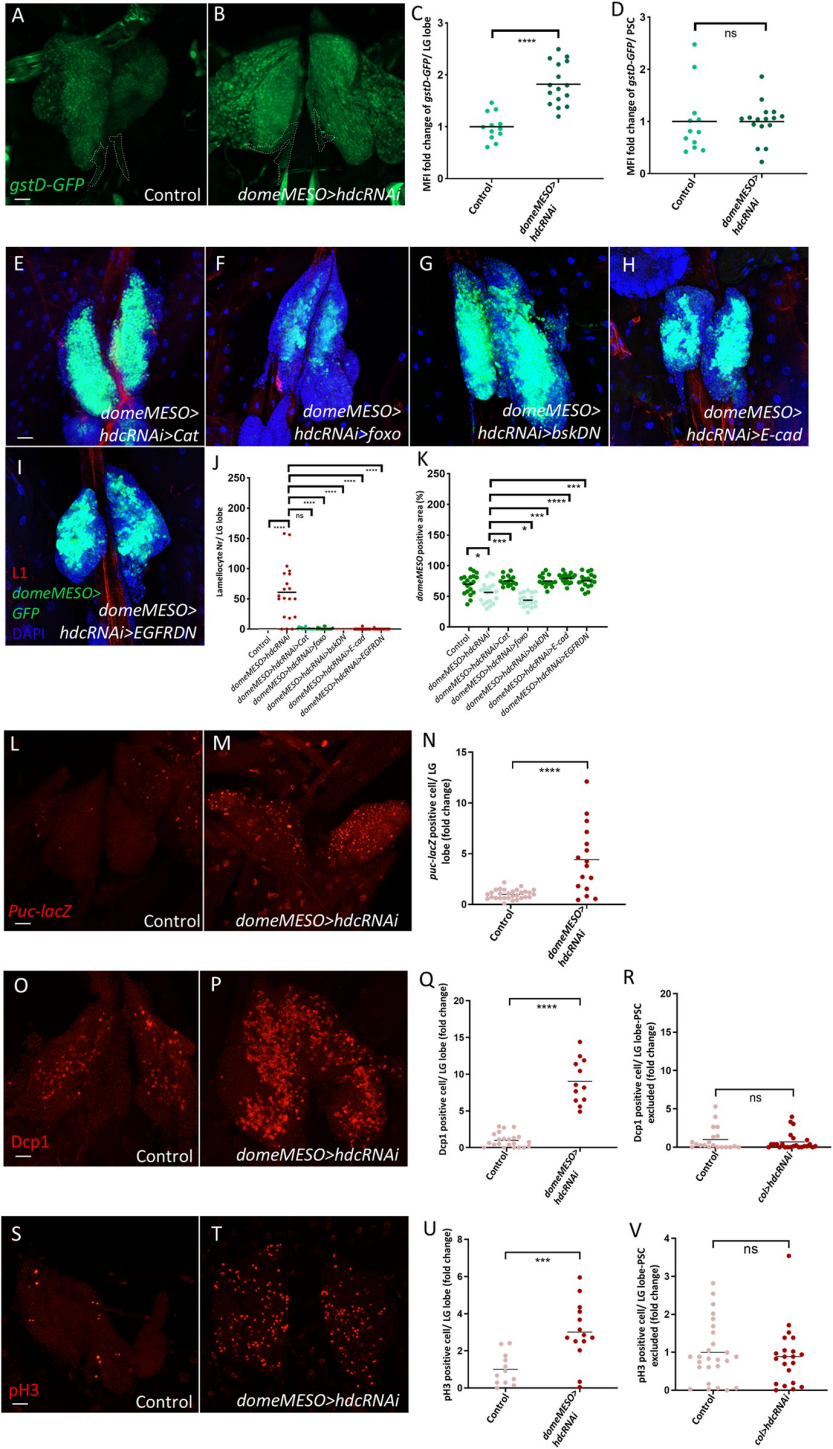
Hdc loss in the MZ causes lamellocyte differentiation through cell-autonomous mechanisms. (A-B) Silencing *hdc* in the MZ leads to higher levels of ROS in the anterior lobes but not in the PSC as visualized by the *gstD-GFP* reporter (*UAS-hdcRNAi/gstD-GFP; domeMESO-GAL4/+*) (n = 16) (B) in comparison to the control (*gstD-GFP/+; domeMESO-GAL4/+*) (n = 12) (A) (green: ROS). *n* refers to the number of lymph glands analyzed. Scale bar: 20 μm. (C-D) Scatter dot plots showing the fold change increase in the MFI of *gstD-GFP* in the anterior lymph gland lobes (average = 1.8 folds) (C) and the indifference in MFI of *gstD-GFP* in the PSC of *domeMESO>hdcRNAi* (*UAS-hdcRNAi/gstD-GFP; domeMESO-GAL4/+*) larvae in comparison to the control (*gstD-GFP/+; domeMESO-GAL4/+*) (D). Each dot in the graphs represents one anterior lobe. Data were analyzed using two-tailed unpaired Student’s t-test, **** *p* ≤ 0.0001, ns: non-significant. (E-I) Overexpression of *Cat* (*UAS-hdcRNAi/UAS-Cat; domeMESO-GAL4*,*UAS-2xEGFP/+*) (n = 16) (E) or *foxo* (*UAS-hdcRNAi/UAS-foxo; domeMESO-GAL4*,*UAS-2xEGFP/+*) (n = 18) (F) or expression of a dominant negative version of *bsk* (*UAS-hdcRNAi/+; domeMESO-GAL4*,*UAS-2xEGFP/UAS-bsk53R*) (n = 14) (G), or overexpression of *E-cad* (*UAS-hdcRNAi/+; domeMESO-GAL4*,*UAS-2xEGFP/UAS-E-cad*) (n = 18) (H) or expression of a dominant negative version *of EGFR* (*UAS-hdcRNAi/UAS-EGFR*.*DN; domeMESO-GAL4*,*UAS-2xEGFP/UAS-EGFR*.*DN*) (n = 18) (I) rescues lamellocyte differentiation in *hdc* silenced background (blue: nuclei, green: MZ, red: lamellocytes). *n* refers to the number of lymph glands analyzed. Scale bar: 20 μm. (J) The number of lamellocytes per lymph gland lobe from the genotypes presented in panels (E-I) in addition to the control (*domeMESO-GAL4*,*UAS-2xEGFP/+*) (n = 20) and *domeMESO>hdcRNAi* (*UAS-hdcRNAi/+; domeMESO-GAL4*,*UAS-2xEGFP/+*) (n = 20). *n* refers to the number of lymph glands analyzed. Each dot in the graph represents one lymph gland lobe. Data were analyzed using ANOVA with Tukey’s test for multiple comparisons, **** *p* ≤ 0.0001, ns: non-significant. (K) The percentage of *domeMESO* positive area per anterior lymph gland lobe from the genotypes presented in panels (E-I) in addition to the control (*domeMESO-GAL4*,*UAS-2xEGFP/+*) (n = 20) and *domeMESO>hdcRNAi* (*UAS-hdcRNAi/+; domeMESO-GAL4*,*UAS-2xEGFP/+*) (n = 20). *n* refers to the number of lymph glands analyzed. Each dot in the graph represents one lymph gland lobe. Data were analyzed using ANOVA with Tukey’s test for multiple comparisons, * *p* ≤ 0.05, *** *p* ≤ 0.001, **** *p* ≤ 0.0001. (L-M) Silencing *hdc in the MZ* increases the number of *puc-lacZ* positive cells (*UAS-hdcRNAi/+; domeMESO-Gal4*,*UAS-2xEGFP/puc-lacZ*) (n = 16) (M) in comparison to the control (*domeMESO-Gal4*,*UAS-2xEGFP/puc-lacZ*) (n = 30) (L) (red: *puc-LacZ*). Scale bar: 20 μm. (N) A scatter dot plot showing fold change increase in *puc-LacZ* cell number (average = 4.4 folds) per anterior lobe of *domeMESO>hdcRNAi* (*UAS-hdcRNAi/+; domeMESO-Gal4*,*UAS-2xEGFP/puc-lacZ*) larvae compared to the control (*domeMESO-Gal4*,*UAS-2xEGFP/puc-lacZ*). Each dot in the graph represents one anterior lobe. Data were analyzed using two-tailed unpaired Student’s t-test, ns: non-significant. (O-P) Silencing *hdc in the MZ* increases the number of Dcp1 positive cells (*UAS-hdcRNAi/+; domeMESO-Gal4*,*UAS-2xEGFP/+*) (n = 12) (P) in comparison to the control (*domeMESO-Gal4*,*UAS-2xEGFP/+*) (n = 22) (O) (red: Dcp1). Scale bar: 20 μm. (Q) A scatter dot plot showing fold change increase in the number of Dcp1 positive cells (average = 9 folds) per anterior lobe of *domeMESO>hdcRNAi* (*UAS-hdcRNAi/+; domeMESO-Gal4*,*UAS-2xEGFP/+*) larvae compared to the control (*domeMESO-Gal4*,*UAS-2xEGFP/+*). Each dot in the graph represents one anterior lobe. Data were analyzed using two-tailed unpaired Student’s t-test, **** *p* ≤ 0.0001. (R) A scatter dot plot showing the insignificant difference in fold change of the number of Dcp1 positive cells (average = 0.7) per anterior lobe (PSC excluded) of *col>hdcRNAi* (*Pcol85-Gal4*,*UAS-2xEGFP UAS-hdcRNAi*) larvae (n = 24) compared to the control (*Pcol85-Gal4*,*UAS-2xEGFP /+*) (n = 20). Each dot in the graph represents one anterior lobe. Data were analyzed using two-tailed unpaired student’s t-test, ns: non-significant. (S-T) Silencing *hdc in the MZ* increases the number of pH3 positive cells (*UAS-hdcRNAi/+; domeMESO-Gal4*,*UAS-2xEGFP/+*) (n = 14) (T) in comparison to the control (*domeMESO-Gal4*,*UAS-2xEGFP/+*) (n = 12) (S) (red: pH3). Scale bar: 20 μm. (U) A scatter dot plot showing fold change increase in the number of pH3 positive cells (average = 3 folds) per anterior lobe of *domeMESO>hdcRNAi* (*UAS-hdcRNAi/+; domeMESO-Gal4*,*UAS-2xEGFP/+*) larvae compared to the control (*domeMESO-Gal4*,*UAS-2xEGFP/+*). Each dot in the graph represents one anterior lobe. Data were analyzed using two-tailed unpaired student’s t-test, *** *p* ≤ 0.001. (V) A scatter dot plot showing the insignificant difference in fold change of the number of pH3 positive cells (average = 0.8) per anterior lobe (PSC excluded) of *col>hdcRNAi* (*Pcol85-Gal4*,*UAS-2xEGFP/UAS-hdcRNAi*) larvae (n = 20) compared to the control (*Pcol85-Gal4*,*UAS-2xEGFP/+*) (n = 26). Each dot in the graph represents one anterior lobe. Data were analyzed using two-tailed unpaired student’s t-test, ns: non-significant.

As *hdc* silencing in the niche resulted in an accumulation of ROS in the PSC, we sought to investigate whether knocking down *hdc* in the MZ would produce a comparable phenotype. The expression of the *gstD-GFP* reporter revealed elevated levels of ROS in *domeMESO>hdcRNAi* medullary zones with no significant difference in ROS levels in the PSC as compared to the control (Fig 5A and 5B, quantified in 5C and 5D). Similar elevation was observed in *hdc*^*Δ84*^ null mutants and was validated using DHE (S6A, S6B and S6D–S6F Fig, quantified in S6C and S6G). In addition, we observed that lamellocyte differentiation and the reduction in MZ size seen in *domeMESO>hdcRNAi* lymph glands was suppressed by overexpression of *Catalase* (Figs 5E and S4C, quantified in 5J, 5K and S4H), suggesting that, similarly to the niche, ROS functions as a signaling molecule in the MZ in response to *hdc* silencing to trigger lamellocyte differentiation. Interestingly, although overexpressing *foxo* in the MZ also significantly decreased lamellocyte numbers in the lymph gland and circulation of *domeMESO>hdcRNAi* animals, it significantly reduced MZ size (Figs 5F and S4D, quantified in 5J, 5K and S4H). This is most probably due to the fact that *foxo* overexpression forces progenitor differentiation into plasmatocytes and crystal cells, as reported in literature [61] and replicated in our experiments (S5A and S5G Fig, quantified in S5D and S5H), and thus preventing their differentiation into lamellocytes. Furthermore, unlike in the niche, we did not observe an induction in *Thor-LacZ* reporter upon depleting Hdc in the MZ (S3M and S3N Fig, quantified in S3O), suggesting a different response for progenitors to Hdc depletion compared to PSC cells. Moreover, we did not observe an upregulation in pAkt levels in the MZ upon *hdc* knockdown (S6K and S6L Fig, quantified in S6M), suggesting that the insulin/mTOR pathway is not involved downstream to *hdc* in the MZ.

As higher ROS levels in the MZ were shown previously to activate the JNK pathway [61,62] which, through lowering the levels of the adherens junction protein E-cadherin (E-cad), trigger their differentiation into effector hemocytes [62], we investigated whether JNK is upregulated in response to *hdc* silencing. By utilizing a LacZ reporter for *puckered* (*puc-LacZ*), a downstream target of JNK and a widely used reporter for its activity [63], we observed higher expression levels in the MZ of *domeMESO>hdcRNAi* larvae compared to controls (Fig 5L, 5M and quantified in 5N). Moreover, blocking JNK signaling or overexpressing *E-cad* efficiently rescued lamellocyte differentiation and the reduction in MZ size seen in *domeMESO>hdcRNAi* larvae (Figs 5G, 5H, S4E, and S4F, quantified in 5J, 5K and S4H), implying that JNK and E-cad act downstream to *hdc* in the MZ.

The EGFR pathway has previously been established to be necessary for the immune response of progenitors against parasitic wasp infestation [15]. Another recent report also showed that the loss of Pointed (Pnt), an EGFR effector, in the MZ impedes progenitor differentiation [28]. For this reason, we aimed to explore whether modulating the EGFR pathway in *domeMESO>hdcRNAi* larvae would rescue their mutant phenotype. Interestingly, we found that blocking EGFR by expressing a dominant negative form also rescued lamellocyte differentiation and restored MZ size to normal in *domeMESO>hdcRNAi* larvae (Figs 5I and S4G, quantified in 5J, 5K and S4H), suggesting that EGFR likely acts downstream to *hdc* in the MZ. Notably, knocking down JNK and EGFR or overexpressing E-cad in the niche did not rescue lamellocyte differentiation in *col>hdcRNAi* larvae (S7A–S7D and S7A’–S7D’ Fig, quantified in S7E and S7E’), supporting the notion that distinct mechanisms regulate progenitor differentiation downstream to *hdc* in the MZ compared to the PSC.

In addition to the effect of *hdc* silencing on progenitor differentiation into lamellocytes, we investigated whether *hdc* depletion in progenitors leads to apoptosis or decreased cell division, similar to our observations in the niche. We indeed observed an increase in Dcp1 positive cell number in *domeMESO>hdcRNAi* lymph glands in comparison to the control (Fig 5O, 5P and quantified in 5Q). Notably, when *hdc* was depleted only in the PSC, the caspase activation outside the niche (MZ and CZ) remained unchanged (Fig 5R), suggesting that this effect of *hdc* loss is cell-autonomous. These results also indicate that *hdc* silencing activates Dcp1, which in turn can lead to apoptosis or possibly effector cell differentiation [64], depending on the cellular context. In addition, we observed an increase in pH3 positive cell number in *domeMESO>hdcRNAi* lymph glands (Fig 5S, 5T and quantified in 5U), a phenotype not observed in *col>hdcRNAi* animals (Fig 5V), further implying that *hdc* silencing may affect cell cycle depending on the cell type.

## Discussion

The *Drosophila* lymph gland provides an excellent model to investigate blood cell progenitor maintenance [7,8,17–20,60]. In this study, we delved further into the previously reported non-cell autonomous function of Hdc in the hematopoietic niche. Although it was documented that PSC-specific silencing of *hdc* leads to the differentiation of lamellocytes that are typically not present in naive larvae [43], the precise mechanism underlying the phenotype was not previously understood. Our findings indicate that Hdc is required in the PSC to prevent the overactivation of the insulin/mTOR pathway. This is underlined by the upregulation of pAkt levels in the niche upon *hdc* silencing, the genetic interaction between *hdc* and *unk* (a previously described partner of Hdc involved in the regulation of the insulin/mTOR pathway [39,40]), the rescue of *col>hdcRNAi* lamellocyte phenotype when knocking down the insulin pathway simultaneously with *hdc* in the niche, and the suppression of lamellocyte differentiation in larvae where the insulin pathway is overactivated (*col>ptenRNAi)* by overexpressing *hdc*. These results are in line with previous observations suggesting that Hdc and Unk bind to the mTOR complex through Raptor to inhibit its activity in the imaginal discs [40], and are consistent with prior studies revealing that the continuous activation of the insulin/mTOR pathway in the niche triggers lamellocyte differentiation in the lymph gland [44,45].

Interestingly, although these studies reported that activation of the insulin/mTOR pathway increases PSC size [44–46], the elevation of niche cell number was not observed in *hdc* mutant larvae or in case of PSC-specific silencing of *hdc* [43]. Here we show that *hdc* silencing causes apoptosis and G2 arrest in the niche, and we demonstrate that inhibiting apoptosis in the niche of *col>hdcRNAi* larvae increases its size, suggesting that cell death caused by *hdc* silencing compensates for the increase of niche cell number caused by the overactivation of the insulin pathway. This agrees with studies showing that *hdc* loss-of-function causes apoptosis in the adult progenitor cells (APCs), as well as in stem cells of the testis and the intestine [37,38,41] and reinforces the link of Hdc to the insulin pathway in the niche.

The continuous activation of the insulin/mTOR pathway increases ribogenesis and protein synthesis, which in turn leads to cellular stress hallmarked by elevated ROS levels [41,45]. We found that three markers of oxidative stress, *gstD-GFP*, DHE and *Thor-lacZ* become active in the PSC when *hdc* is silenced. The alleviation of the hematopoietic phenotype resulting from *hdc* silencing in the niche through the overexpression of *Catalase* and *foxo* suggests that ROS plays a key role in lamellocyte differentiation when *hdc* function is compromised. Although in our experiments *Thor-lacZ* indicated elevated Foxo function in response to *hdc* silencing, this endogenous activity seems to be insufficient to compensate for elevated ROS levels. In line with the above findings, we observed that knocking down *foxo* enhanced *hdc* phenotype, resulting in higher lamellocyte numbers. All these results are in accordance with previous data showing that higher ROS levels in the niche trigger MZ progenitor differentiation [45,51].

As demonstrated in our previous study, lamellocyte differentiation in *col>hdcRNAi* larvae could be rescued by overexpressing *hh* and *dpp* in the niche [43]. In light of our current findings, it is possible that cellular stress in the niche cells invoked by *hdc* loss compromises their ability to produce or secrete sufficient amounts of Hh and Dpp to the progenitors, however this crosstalk between regulatory pathways remains to be investigated.

In addition to characterizing the non-cell-autonomous function of Hdc in the PSC, we have identified a novel cell-autonomous role for Hdc in the MZ progenitors to suppress their differentiation into lamellocytes. Interestingly, a specific subpopulation of the progenitors, the distal progenitors, seem to be sensitive for Hdc depletion, as silencing *hdc* in core progenitors or intermediate progenitors does not lead to lamellocyte differentiation. This observation is not the first of its kind, as it was shown earlier that progenitor subpopulations respond differently to the manipulation of certain signaling pathways [25,28], and it further underlines the heterogeneity of the lymph gland progenitors. Moreover, our results showing a reduction in the number of crystal cells in the lymph gland upon *hdc* depletion in the niche or the MZ, agree with earlier data suggesting that progenitors prioritize lamellocyte fate over differentiation into crystal cells [29].

**Fig 6:**
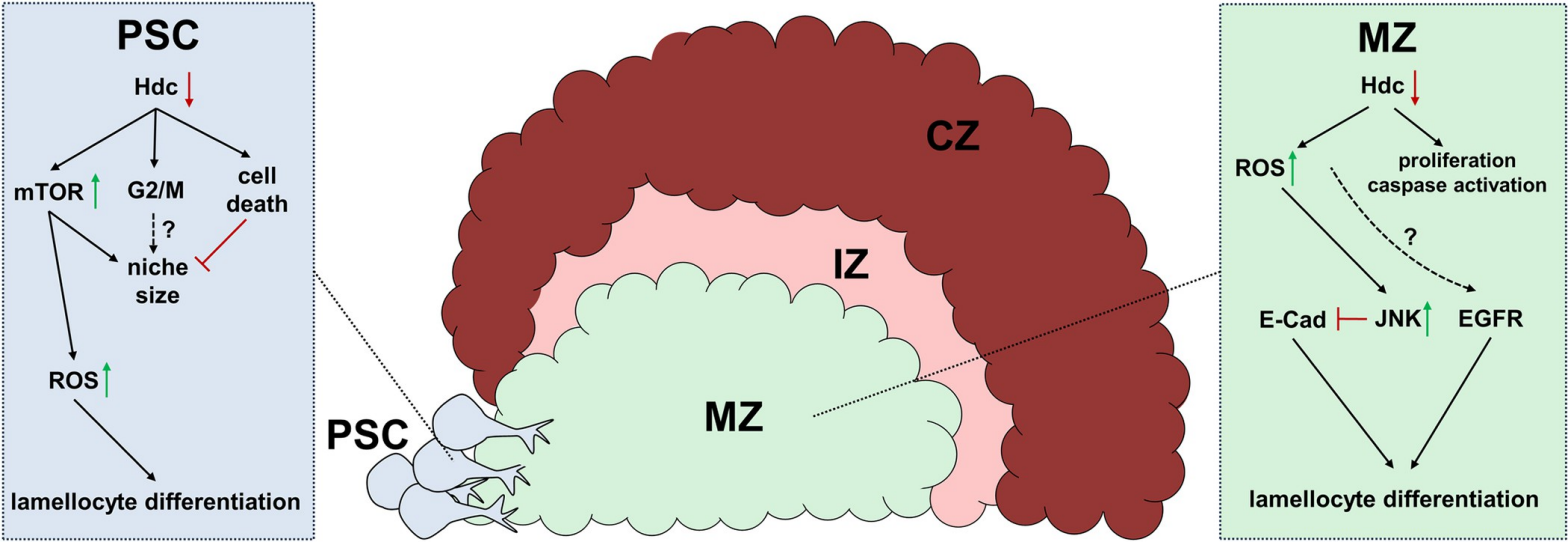
A graphical summary of the dual role for Hdc in the lymph gland of the *Drosophila melanogaster* larva. In the hematopoietic niche, silencing *hdc* leads to overactivation of the insulin/mTOR pathway. The consequent elevation of ROS levels results in impaired progenitor maintenance and differentiation of lamellocytes. The increase in PSC size as a result of insulin/mTOR activation is compensated by cell death and possibly by G2/M cell cycle arrest caused by *hdc* loss-of-function. Although *hdc* silencing also leads to the elevation of ROS levels in the medullary zone of the lymph gland, the insulin/mTOR pathway is not involved in the resulting phenotype. Instead, the JNK pathway, which was previously shown to function downstream to ROS in the MZ, plays a major role. The activation of the JNK pathway downregulates E-cadherin levels thereby damaging cell-cell connections, which leads to lamellocyte differentiation. Although our genetic interaction studies suggest that EGFR functions downstream to Hdc as well, how it is activated by elevated ROS levels, remains to be investigated. In addition to lamellocyte differentiation, silencing *hdc* in the MZ leads to higher caspase activity and cell proliferation in the lymph gland.

Although silencing *hdc* in both the PSC and MZ leads to higher ROS levels, yet we found that both zones respond differently to Hdc depletion. For example, *Thor-lacZ* reporter which is induced in the PSC of *col>hdcRNAi* larvae, was not upregulated in the medullary zone of *domeMESO>hdcRNAi* larvae. Moreover, we found that distinct signaling pathways are implicated downstream to *hdc* in the MZ than in the PSC. For instance, the *hdc* knockdown phenotype in the MZ is not mediated by the insulin/mTOR pathway, but rather the activity of the JNK pathway, and possibly EGFR signaling and E-cad (Fig 6). These results further support the involvement of ROS in response to *hdc* loss in the progenitors, as elevated ROS levels in these cells were described to cause their premature differentiation through activating the JNK pathway and lowering the levels of E-cad junctions, and suggest a possible direct role not shown previously for the EGFR pathway downstream to ROS in the MZ [62].

Interestingly, apart from triggering their differentiation into lamellocytes, silencing *hdc* in the progenitors appears to induce caspase activation, similarly to the niche. However, based on a recent report, caspase activation in progenitors could also be linked to their differentiation rather than apoptosis [64]. Moreover, we found that depleting Hdc in the MZ leads to increased cell division, an opposing effect to what was observed in the niche. This is similar to the dual effect of Transforming Growth Factor-beta (TGF-β) in human cancers, where it promotes cell proliferation in mesenchymal cells, while in epithelial cells, it blocks cell division by inducing cell cycle arrest [65].

Taken together, our data suggest that Hdc suppresses lamellocyte differentiation both non-cell-autonomously in the PSC and in a cell-autonomous fashion in the MZ, however, this function is carried out by distinct downstream mechanisms in the signaling niche and progenitor cells.

What further underlines the role of Hdc in hemocyte progenitor fate determination is the fact that *hdc* expression is downregulated in hemocyte progenitors as they prepare to differentiate at the end of larval stages [43], as well as after parasitic wasp infestation. Since it was previously reported that wasp infestation induces ROS production in the lymph gland, which is necessary for lamellocyte differentiation [15], it is tempting to speculate if this finding can be mechanistically linked to the downregulation of *hdc*, and possibly its partners post-parasitosis. Furthermore, our findings fully agree with early reports stating that *hdc* expression ceases in the imaginal cells when they differentiate into adult tissues [35], and suggest that Hdc plays a general role in suppressing early loss of progenitors and imaginal cells in the larva, possibly through the maintenance of a basal level of ROS in those tissues. Since the human orthologue of Hdc, HECA, was shown to be a tumor suppressor in several tumor models [66–68], our results on how Hdc regulates progenitor differentiation in *Drosophila* may implicate mechanisms potentially relevant in combating HECA-related cancer types.

## Materials and methods

### *Drosophila* stocks and maintenance

The following *Drosophila* stocks were used: *w*^*1118*^ (BDSC#5905), *Pcol85-Gal4/CyO*,*GFP* [20], *Pcol85-Gal4*,*UAS-hdcRNAi/CyO*,*GFP* [43], *UAS-unkRNAi* (BDSC#57026), *UAS-Pi3K92E*.*CAAX* (BDSC#8294), *UAS-PtenRNAi* (BDSC#8550), *UAS-AktRNAi* (BDSC#31701), *UAS-raptorRNAi* (BDSC#31528), *Pcol85-Gal4/CyO*,*GFP; UAS-PtenRNAi* (generated by combining *Pcol85-Gal4* with *UAS-PtenRNAi*), *UAS-hdc*.*S* (a gift from Christos Samakovlis), *Pcol85-Gal4*,*UAS-2xEGFP/SM6b* (generated by recombination of *Pcol85-Gal4* with *UAS-2xEGFP* (BDSC#6874)), *Pcol85-Gal4*,*UAS-hdcRNAi/CyO*,*GFP;UAS-2xEGFP* (generated by recombination *Pcol85-Gal4*,*UAS-hdcRNAi/CyO*,*GFP* with *UAS-2xEGFP* (BDSC#6658)), *UAS-FlyFUCCI* (BDSC#55122 [49]), *UAS-p35* (BDSC#5073), *gstD-GFP* (a gift from Lolitika Mandal [52]), *puc-lacZ* (BDSC#11173) [63], *Thor-lacZ* (BDSC#9558), *UAS-Cat* (BDSC#24621), *UAS-foxo* (BDSC#9575), *UAS-foxoRNAi* (BDSC#25997), *UAS-spiRNAi* (VDRC#v103817), *UAS-mCD8*::*GFP; UAS-hdc*^*19*^*-Gal4/TM6Tb* (generated from combining *hdc*^*19*^*-Gal4* [43] with *UAS-mCD8*::*GFP* (a gift from József Mihály), *Hml*:*DsRed* [69], *domeMESO-GAL4*,*UAS-2xEGFP/TM6* [60], *UAS-hdcRNAi* (VDRC#v45069), *Tep4-Gal4* (a gift from Gregory D. Longmore [34]),*CHIZ-Gal4/CyO*,*GFP* (a gift from Gregory D. Longmore, the *CyO* balancer was changed to *CyO*,*GFP* [27]), *gstD-GFP*, *domeMESO-Gal4/TM6TB* (generated by combining *gstD-GFP* with *domeMESO-Gal4*), *gstD-GFP; hdc*^*Δ84*^*/TM6Tb* (generated by combining *gstD-GFP* with *hdc*^*Δ84*^ [43]), *hdcRNAi/CyO*,*GFP; domeMESO-GAL4*,*UAS-2xEGFP/TM6*,*Tb* (generated from combining *UAS-hdcRNAi* (VDRC#v45069) with *domeMESO-GAL4*,*UAS-2xEGFP*), *UAS-bskDN* (BDSC#9311), *UAS-EgfrDN* (BDSC#5364), *UAS-E-Cad* (a gift from Gregory D. Longmore). The flies were kept on a standard cornmeal-yeast media at 25°C. All crosses were performed at 25°C.

### Antibodies and reagents

The following primary antibodies were used: mouse anti-L1 and mouse anti-P1 (1/10, a gift from István Andó) [9], mouse anti-Col (1/100, a gift from Michèle Crozatier) [20], mouse anti-LacZ (1:100, DSHB 40-1a), mouse anti-C1 (HC12F6) (a gift from Tina Trenczek), rabbit anti-pAkt (1:200, Cell Signaling Technology, CatNo. 4060), rabbit anti-Dcp1 (1:100, cell signaling technology, CatNo.9578), rabbit anti-pH3 (1:200, Cell Signaling Technology CatNo. 3642S). Secondary antibodies were: Goat anti-Rabbit Alexa Fluor 568 (1:1000, Thermo Fisher Scientific, CatNo. A^-11011^), Goat anti-Mouse Alexa Fluor 568 (1:1000, Thermo Fisher Scientific, CatNo. A-11004), Goat anti-Mouse Alexa Fluor Plus 488 (1:1000, Thermo Fisher Scientific, CatNo. A32723), Rabbit anti-Mouse Alexa Fluor 647 (1:1000, Thermo Fisher Scientific, CatNo. A-21239). Nuclei were visualized with DAPI (Sigma-Aldrich).

### Immunostaining, imaging and processing of lymph gland samples

Lymph glands were dissected and stained as described by Varga et al. (2019) [43]. DHE (Thermo Fisher Scientific, CatNo. D23107) staining was performed according to the protocol described by Evans et al. (2014) [54]. The sample numbers indicated in the figure legends always originate from three independent experiments for each genotype.

For each lymph gland, Z-stacks of 10 slices were captured using 20× objective in the Zeiss LSM800 confocal microscope. Images are displayed as the maximum intensity projection of the Z-stacks, after brightness/contrast was adjusted using the ImageJ/Fiji (US National Institutes of Health, Bethesda, MD, USA) image processing software. Images from each experiment and the appropriate control were taken using the same microscope settings. For quantifying lamellocyte (L1 positive cell) numbers in lymph glands, the number of DAPI nuclei that are L1 positive in each lymph gland lobe were counted manually using the multi-point tool in ImageJ/Fiji. PSC cell number (PSC size) was calculated as the number of DAPI nuclei that are positive for *col>GFP* or Col antibody. The number of pH3/Dcp1/Thor-lacZ/Puc-lacZ positive cells were also counted manually using the multi-point tool in ImageJ/Fiji. PSC cells in the G2/M phase were calculated as the number of PSC cells that are both green and red and represented in percentage to the total number of PSC cells. The size of P1 positive (plasmatocyte) area or MZ (*domeMESO>GFP* positive) area in an anterior lobe was measured using Analyze>Measure>Area in ImageJ/Fiji, and the percentage to the size of the anterior lobe was then calculated. Crystal cells in lymph glands (C1 positive cells) were counted manually using the multi-point tool in ImageJ/Fiji, and crystal cell index was calculated as the number of crystal cells in the lobe/size of the lobe as measured using Analyze>Measure>Area in ImageJ/Fiji. For assessing the fluorescence intensity of *hdc>GFP*, *gstD-GFP*,*Thor-LacZ*, DHE, or pAkt, the studied area was selected using the free hand selection tool and the mean fluorescence intensity (MFI) was measured using Analyze>Measure>Mean gray value in ImageJ/Fiji, and the MFI was shown as fold change in comparison to the average MFI in the control of each experiment.

### Immunostaining, imaging and counting of circulating hemocytes

Circulating hemocytes from single larvae were prepared and stained as described by Varga et al. (2019) [43]. Images of the samples were taken using 10x objective in Zeiss Axio Imager Z1 fluorescence microscope. Nuclei were counted automatically using the ‘cellcounter’ macro in ImageJ/Fiji software. Lamellocytes (L1 positive cells) were counted manually using the multi-point tool in ImageJ/Fiji, and the percentage of lamellocytes to the number of nuclei was calculated. A minimum of 100 nuclei were counted from each larva.

### Wasp infestation

In two separate vials, 10 *white* virgins and 10 *hdc>GFP* (*UAS-mCD8*::*GFP; hdc*^*19*^*-Gal4*) males were left to mate for 24 hours. The next day, the females of each cross were transferred to a new vial to lay eggs for 4 hours. At 72 hours after egg laying, 25 female *Leptopilina boulardi* G486 parasitic wasps were added to one of the two vials and left to infest the larvae for 4 hours at 25°C, while the other vial was left at 25°C without infestation to serve as a control. 16 hours post infestation (hpi), infested larvae were selected based on the melanized injury site caused by the oviposition. Lymph glands were dissected from infested, as well as from naive control larvae.

### Data analysis

All quantitative analysis of data and graphs were done using GraphPad Prism 8. For data consisting of two groups, two-tailed unpaired Student’s t-test was used, while for data consisting of more than two groups, analysis of variance (ANOVA) with Tukey’s test for multiple comparisons was utilized. Values of *p* < 0.05 were accepted as significant (* *p* ≤ 0.05, ** *p* ≤ 0.01, *** *p* ≤ 0.001, **** *p* ≤ 0.0001, ns: non-significant).

## Supporting information

S1 Fig Circulation samples from the interaction between *hdc* and the insulin pathway in the niche. (A-F) Lamellocytes (red) are absent in the circulation of control larvae (*Pcol85-Gal4/+*) (0.08% (n = 43)) (A), but can be detected when *hdc* is silenced in the PSC (*Pcol85-Gal4*,*UAS-hdcRNAi/+*) (1.9% (n = 53)) (B), its partner *unk* is silenced (*Pcol85-Gal4/UAS-unkRNAi)* (1.1% (n = 44)) (C), both *hdc* and *unk* are silenced together (*Pcol85-Gal4*,*UAS-hdcRNAi/unkRNAi*) (3.2% (n = 48)) (D), the insulin/mTOR pathway is activated in the PSC by expressing *Pi3KCa* (UAS-*Pi3K92E*.*CAAX /+; Pcol85-Gal4/+*) (1.3% (n = 39)) (E), or silencing *Pten* (*Pcol85-Gal4/+; UAS-PtenRNAi/+*) (1.4% (n = 51)) (F). (G-H) The number of lamellocytes in the circulation of *col>hdcRNAi* larvae is reduced when simultaneously *Akt* (*Pcol85-Gal4*,*UAS-hdcRNAi/+; UAS-AktRNAi/+*) (0.08% (n = 41)) (G) or *raptor* (*Pcol85-Gal4*,*UAS-hdcRNAi/+; UAS-raptorRNAi/+*) (0.2% (n = 34)) is silenced (H). (I) Overexpression of *hdc* reduces lamellocyte numbers in the circulation of *col>PtenRNAi* larvae (*Pcol85-Gal4/+; UAS-PtenRNAi/UAS-hdc*.*S*) (0.14% (n = 36)). *n* refers to the number of larvae analyzed. Nuclei are visualized by DAPI (blue). Scale bar:20 μm. (J) A scatter dot plot showing the percentage of lamellocytes in the circulation of larvae from the genotypes presented in panels (A-I). Each dot in the graph represents a single larva. Data were analyzed using ANOVA with Tukey’s test for multiple comparisons, * p ≤ 0.05, ** *p* ≤ 0.01, *** *p* ≤ 0.001, **** *p* ≤ 0.0001.
(TIF)

S2 Fig Supplementary data on the link between Hdc and ROS in the PSC. (A-C) Silencing *hdc* results in higher ROS levels in the PSC (*col>GFP* positive area) as shown by the oxidation of the ROS indicator DHE dye (red) (*Pcol85-Gal4*,*UAS-2xEGFP/UAS-hdcRNAi*) (n = 14) (B), which is not observed in the control (*Pcol85-Gal4*,*UAS-2xEGFP/+*) (n = 14) (A) or when *Akt* is silenced simultaneously with *hdc* in the PSC (*Pcol85-Gal4*,*UAS-hdcRNAi/+; UAS-AktRNAi/UAS-2xEGFP*) (n = 8) (C). *n* refers to the number of lymph gland lobes analyzed. Scale bar: 20 μm. (D) A scatter dot plot showing the mean fluorescence intensity (MFI) of DHE (represented in fold change in comparison to the control) from the genotypes presented in the panels (A-C). Each dot in the graph represents a PSC from one lobe. Data were analyzed using ANOVA with Tukey’s test for multiple comparisons, **** *p* ≤ 0.0001, ns: non-significant. (E-G) Overexpressing Cat (*Pcol85-Gal4*,*UAS-hdcRNAi/UAS-Cat; UAS-2xEGFP/+*) (average number of PSC cells = 35, n = 14) (F) or *foxo* (*Pcol85-Gal4*,*UAS-hdcRNAi/UAS-foxo; UAS-2xEGFP/+*) (average number of PSC cells = 29, n = 14) (G) did not change the size of the PSC (number of col>GFP positive cells) of *col>hdcRNAi* (*Pcol85-Gal4*,*UAS-hdcRNAi/+; UAS-2xEGFP/+*) (average number of PSC cells = 27, n = 10) larvae (E) (blue: nuclei, green: PSC). *n* refers to the number of lymph gland lobes analyzed. Scale bar: 20 μm. (H) A scatter dot plot showing PSC cell number in larvae from the genotypes presented in panels (E-G). Each dot in the graph represents a PSC from one lymph gland lobe. Data were analyzed using ANOVA with Tukey’s test for multiple comparisons, ns: non-significant.
(TIF)

S3 Fig Circulation samples from the interaction between *hdc* and cellular stress modulators in the PSC. (A-C) Overexpression of *Cat* (*Pcol85-Gal4*,*UAS-hdcRNAi/UAS-Cat*) (0.25% (n = 40)) (B) or *foxo* (*Pcol85-Gal4*,*UAS-hdcRNAi/UAS-foxo*) (0.08% (n = 51)) (C) rescues lamellocyte differentiation in *hdc* silenced larvae (*Pcol85-Gal4*,*UAS-hdcRNAi/+*) (3.3% (n = 77)) (A). (D-E) Silencing *foxo* simultaneously with *hdc* (*Pcol85-Gal4*,*UAS-hdcRNAi/+; UAS-foxoRNAi/+*) enhances the lamellocyte differentiation phenotype associated with *hdc* silencing (6% (n = 58)) (E), while silencing *foxo* alone in the niche does not lead to lamellocyte differentiation (*Pcol85-Gal4/+; UAS-foxoRNAi/+*) (0.19% (n = 15)) (D). (F) Silencing *spi* in the niche does not affect lamellocyte numbers in the circulation of *col>hdcRNAi* larvae (*Pcol85-Gal4*,*UAS-hdcRNAi/UAS-spiRNAi*) (2.8% (n = 24)) (blue: nuclei, red: lamellocytes). *n* refers to the number of larvae analyzed. Scale bar: 20 μm. (E) A scatter dot plot quantifying lamellocyte numbers in larvae from the genotypes presented in panels (A-F). Each dot in the graph represents a single larva. Data were analyzed using ANOVA with Tukey’s test for multiple comparisons, ** *p* ≤ 0.01, *** *p* ≤ 0.001, **** *p* ≤ 0.0001, ns: non-significant.
(TIF)

S4 Fig Circulation samples from the genetic interactions of Hdc in the MZ. (A-B) Silencing *hdc* in the MZ leads to the appearance of lamellocytes in the circulation (*UAS-hdcRNAi/+; domeMESO-GAL4*,*UAS-2xEGFP/+*) (3.7% (n = 67)) (B), which are normally not present in the control (*domeMESO-GAL4*,*UAS-2xEGFP/+*) (0.04% (n = 33)) (A). (C-G) Overexpression of *Cat* (*UAS-hdcRNAi/UAS-Cat; domeMESO-GAL4*,*UAS-2xEGFP/+*) (0.2% (n = 24)) (C) or *foxo* (*UAS-hdcRNAi/UAS-foxo; domeMESO-GAL4*,*UAS-2xEGFP/+*) (0% (n = 22)) (D) or a dominant negative form of *bsk* (*UAS-hdcRNAi/+; domeMESO-GAL4*,*UAS-2xEGFP/UAS-bsk53R*) (0.01% (n = 26)) (E), *E-cad* (*UAS-hdcRNAi/+; domeMESO-GAL4*,*UAS-2xEGFP/UAS-E-cad*) (0.03% (n = 19)) (F) or a dominant negative form of *EGFR* (*UAS-hdcRNAi/UAS-EGFR*.*DN; domeMESO-GAL4*,*UAS-2xEGFP/UAS-EGFR*.*DN*) (0% (n = 26)) (G) is able to rescue *hdc* lamellocyte phenotype (blue: nuclei, red: lamellocytes). *n* refers to the number of larvae analyzed. Scale bar: 20 μm. (H) A scatter dot plot showing percentage of lamellocytes in the circulation of larvae from the genotypes presented in panels (A-G). Each dot in the graph represents one single larva. Data were analyzed using ANOVA with Tukey’s test for multiple comparisons, *** *p* ≤ 0.001, **** *p* ≤ 0.0001.
(TIF)

S5 Fig The effect of Hdc depletion in the MZ and the PSC on plasmatocytes and crystal cells. (A-C) Silencing *hdc* in the MZ (*UAS-hdcRNAi/+; domeMESO-GAL4*,*UAS-2xEGFP/+*) does not affect P1 positive (plasmatocyte) area percentage per anterior lobe (average = 33%, number of lobes = 6) (B), while overexpressing *foxo* in *dome>hdcRNAi* larvae (*UAS-hdcRNAi/UAS-foxo; domeMESO-GAL4*,*UAS-2xEGFP/+*) significantly increases it (average = 79%, number of lobes = 10) (C) in comparison to the control (*domeMESO-GAL4*,*UAS-2xEGFP/+*) (average = 41%, number of lobes = 6) (A) (blue: nuclei, green: MZ, red: plasmatocytes). Scale bar: 20 μm. (D) A scatter dot plot showing P1 positive (plasmatocyte) area percentage per anterior lobe from the genotypes in the panels (A-C). Each dot in the graph represents one anterior lobe. Data were analyzed using ANOVA with Tukey’s test for multiple comparisons, **** *p* ≤ 0.0001, ns: non-significant. (E-G) Silencing *hdc* in the MZ (*UAS-hdcRNAi/+; domeMESO-GAL4*,*UAS-2xEGFP/+*) significantly reduces the crystal cell index (average = 3.2, number of lobes = 22) (F), while overexpressing *foxo* in *dome>hdcRNAi* larvae (*UAS-hdcRNAi/UAS-foxo; domeMESO-GAL4*,*UAS-2xEGFP/+*) significantly increases it (average = 9.7, number of lobes = 8) (G) in comparison to the control (*domeMESO-GAL4*,*UAS-2xEGFP/+*) (average = 5.4, number of lobes = 28) (E) (blue: nuclei, green: MZ, red: crystal cells). Scale bar: 20 μm. (H) A scatter dot plot quantifying crystal cell index from the genotypes in the panels (E-G). Each dot in the graph represents one anterior lobe. Data were analyzed using ANOVA with Tukey’s test for multiple comparisons, * p ≤ 0.05, ** *p* ≤ 0.01. (I-J) Silencing *hdc* in the PSC (*UAS-hdcRNAi/+; domeMESO-GAL4*,*UAS-2xEGFP/+*) does not affect P1 positive (plasmatocyte) area percentage per anterior lobe (*Pcol85-Gal4*,*UAS-2xEGFP/UAS-hdcRNAi*) (average = 47%, number of lobes = 10) (J), in comparison to the control (*Pcol85-Gal4*,*UAS-2xEGFP/+*) (average = 50%, number of lobes = 14) (I) (blue: nuclei, green: PSC, red: plasmatocytes). Scale bar: 20 μm. (K) A scatter dot plot showing P1 positive (plasmatocyte) area percentage per anterior lobe from the genotypes in the panels (I-J). Each dot in the graph represents one anterior lobe. Data were analyzed using two-tailed unpaired Student’s t-test, ns: non-significant. (L-M) Silencing *hdc* in the PSC (*Pcol85-Gal4*,*UAS-2xEGFP/UAS-hdcRNAi*) significantly reduces the crystal cell index (average = 2.4, number of lobes = 18) (M), in comparison to the control (*Pcol85-Gal4*,*UAS-2xEGFP/+*) (average = 5.6, number of lobes = 14) (L) (blue: nuclei, green: PSC, red: crystal cells). Scale bar: 20 μm. (N) A scatter dot plot quantifying crystal cell index from the genotypes in the panels (L-M). Each dot in the graph represents one anterior lobe. Data were analyzed using two-tailed unpaired Student’s t-test, ** *p* ≤ 0.01.
(TIF)

S6 Fig *hdc* depletion in progenitors triggers ROS production, but does not activate Foxo and insulin/mTOR. (A-B) *hdc*^*Δ84*^ null mutants (*gstD-GFP; hdc*^*Δ84*^) show higher *gstD-GFP* induction (n = 16) (B) in comparison to the control (*gstD-GFP*) (n = 10) (A) (green: *gstD-GFP*). *n* refers to the number of lymph gland lobes analyzed. Scale bar: 20 μm. (C) A scatter dot plot showing the fold change increase (average = 2.7 folds) in the MFI of *gstD-GFP* in the anterior lymph gland lobes of *hdc*^*Δ84*^ null larvae (*gstD-GFP; hdc*^*Δ84*^) in comparison to the control (*gstD-GFP*). Each dot in the graph represents one anterior lobe. Data were analyzed using two-tailed unpaired Student’s t-test, **** *p* ≤ 0.0001. (D-F) *domeMESO>hdcRNAi* larvae (n = 12) (*UAS-hdcRNAi/+; domeMESO-Gal4/+*) (E) and *hdc*^*Δ84*^ null mutants (n = 14) (*hdc*^*Δ84*^) (F) show higher DHE florescence in comparison to the control (*w*^*118*^) (n = 22) (D) (red: DHE). *n* refers to the number of lymph gland lobes analyzed. Scale bar: 20 μm. (G) A scatter dot plot showing the fold change increase in the MFI of DHE in the anterior lymph gland lobes of *domeMESO>hdcRNAi* (average = 1.7 folds) and *hdc*^*Δ84*^ null larvae (average = 2 folds) in comparison to the control. Each dot in the graph represents one anterior lobe. Data were analyzed using ANOVA with Tukey’s test for multiple comparisons, *** *p* ≤ 0.001, **** *p* ≤ 0.0001. (H-I) Silencing *hdc* does not affect the transcription of *Thor* as detected by an anti-lacZ staining for the *Thor-lacZ* reporter (*UAS-hdcRNAi/+; domeMESO-Gal4*,*UAS-2xEGFP/Thor-lacZ*) (n = 6) (I) in comparison to the control (*domeMESO-Gal4*,*UAS-2xEGFP/Thor-lacZ*) (n = 6) (H) (red: Thor-LacZ). Scale bar: 20 μm. (J) A scatter dot plot showing fold change in MFI of Thor-LacZ per anterior lobe of (*UAS-hdcRNAi/+; domeMESO-Gal4/Thor-lacZ*) larvae (average = 0.8) compared to the control (*domeMESO-Gal4/Thor-lacZ*) (average = 1). Each dot in the graph represents one anterior lobe. Data were analyzed using two-tailed unpaired Student’s t-test, ns: non-significant. (K-L) Silencing *hdc* does not affect the levels of pAkt in the lymph gland (*UAS-hdcRNAi/+; domeMESO-Gal4*,*UAS-2xEGFP/+*) (n = 8) (L) in comparison to the control (*domeMESO-Gal4*,*UAS-2xEGFP/+*) (n = 8) (K) (red: pAkt). Scale bar: 20 μm. (M) A scatter dot plot showing fold change in MFI of pAkt per anterior lobe of *domeMESO>hdcRNAi* (*UAS-hdcRNAi/+; domeMESO-Gal4*,*UAS-2xEGFP/+*) (average = 0.9) compared to the control (*domeMESO-Gal4*,*UAS-2xEGFP/+*) (average = 1). Each dot in the graph represents one anterior lobe. Data were analyzed using two-tailed unpaired Student’s t-test, ns: non-significant.
(TIF)

S7 Fig Hdc does not interact with JNK, E-cad and EGFR in the PSC. (A-D) Expressing a dominant negative version of *bsk* (*Pcol85-Gal4*,*UAS-hdcRNAi/+; UAS-bsk53R/+*) (n = 18) (B), or overexpressing *E-cad* (*Pcol85-Gal4*,*UAS-hdcRNAi/+; UAS-E-cad/+*) (n = 12) (C) or a dominant negative version of EGFR (*Pcol85-Gal4*,*UAS-hdcRNAi/UAS-EGFR*.*DN; UAS-EGFR*.*DN/+*) (n = 16) (D) or does not affect lamellocyte differentiation in the lymph glands of *col*>*hdcRNAi* larvae (*Pcol85-Gal4*,*UAS-hdcRNAi/+*) (n = 16) (A) (blue: nuclei, red: lamellocytes). *n* refers to the number of lymph gland lobes analyzed. Scale bar: 20 μm. (E) The number of lamellocytes per lymph gland lobe in the genotypes presented in panels (A-D). Each dot in the graph represents one lymph gland lobe. Data were analyzed using ANOVA with Tukey’s test for multiple comparisons, ns: non-significant. (A’-D’) Expressing a dominant negative version of *bsk* (*Pcol85-Gal4*,*UAS-hdcRNAi/+; UAS-bsk53R/+*) (2.3% (n = 21)) (B’), or overexpressing *E-cad* (*Pcol85-Gal4*,*UAS-hdcRNAi/+; UAS-E-cad/+*) (1% (n = 19)) (C’) or a dominant negative version of EGFR (*Pcol85-Gal4*,*UAS-hdcRNAi/UAS-EGFR*.*DN; UAS-EGFR*.*DN/+*) (1.3% (n = 25)) (D’) does not affect lamellocyte differentiation in the circulation of col>*hdcRNAi* larvae (*Pcol85-Gal4*,*UAS-hdcRNAi/+*) (1.3% (n = 57)) (A’). (blue: nuclei, red: lamellocytes). *n* refers to the number of larvae analyzed. Scale bar: 20 μm. (E’) A scatter dot plot quantifying lamellocyte numbers in larvae from the genotypes presented in panels (A’-D’). Each dot in the graph represents a single larva. Data were analyzed using ANOVA with Tukey’s test for multiple comparisons, ns: non-significant.
(TIF)

S1 Data Source data. (XLSX)

S1 File Graphical abstract. (TIF)
