# Supplementary figures and images for: Dual role for Headcase in hemocyte progenitor fate determination in Drosophila melanogaster
Source: PLoS Genet. 2024 Oct 28;20(10):e1011448. doi: 10.1371/journal.pgen.1011448 (PMC11515969; doi:10.1371/journal.pgen.1011448)

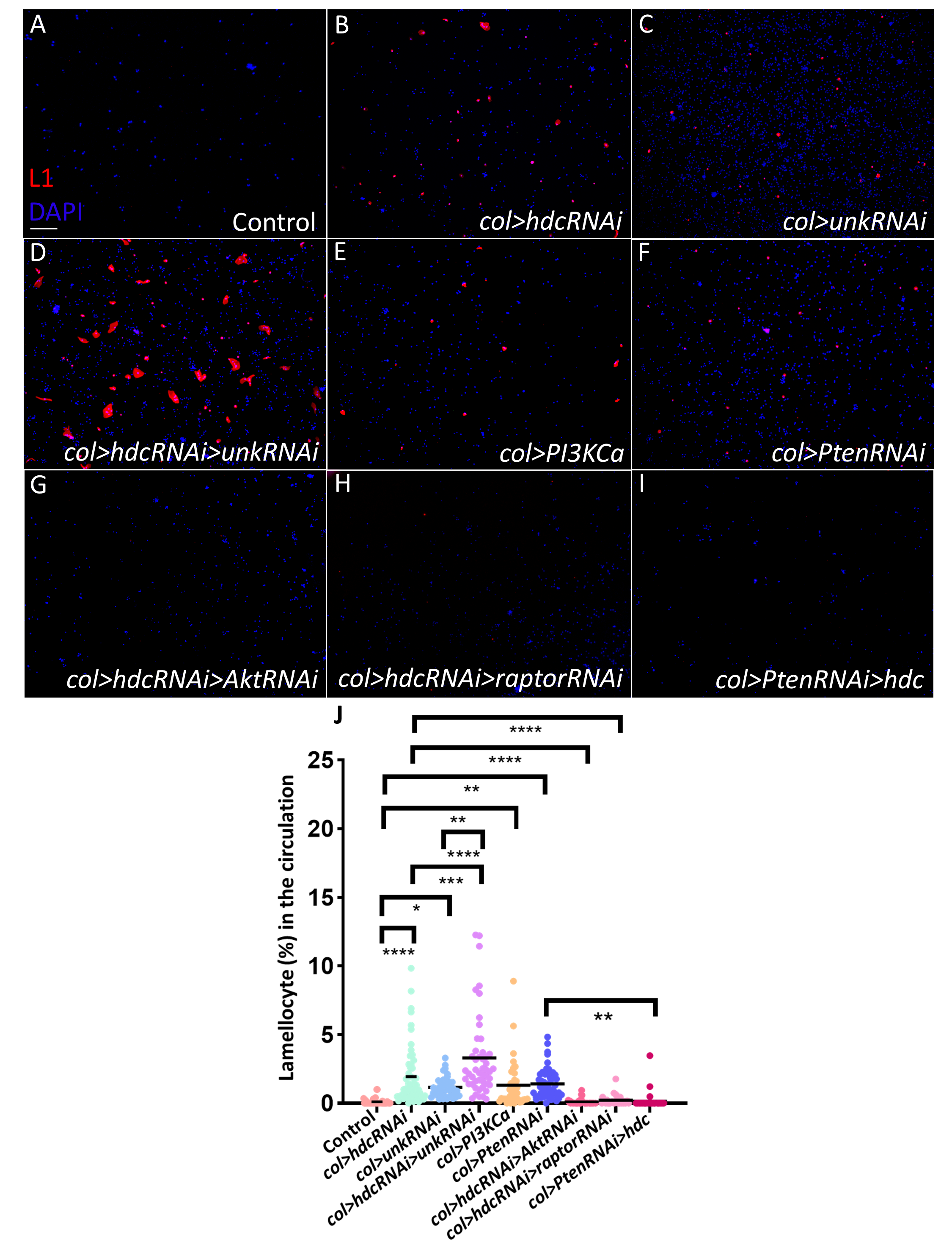

Supplement: S1 Fig — (A-F) Lamellocytes (red) are absent in the circulation of control larvae (Pcol85-Gal4/+) (0.08% (n = 43)) (A), but can be detected when hdc is silenced in the PSC (Pcol85-Gal4,UAS-hdcRNAi/+) (1.9% (n = 53)) (B), its partner unk is silenced (Pcol85-Gal4/UAS-unkRNAi) (1.1% (n = 44)) (C), both hdc and unk are silenced together (Pcol85-Gal4,UAS-hdcRNAi/unkRNAi) (3.2% (n = 48)) (D), the insulin/mTOR pathway is activated in the PSC by expressing Pi3KCa (UAS-Pi3K92E.CAAX /+; Pcol85-Gal4/+) (1.3% (n = 39)) (E), or silencing Pten (Pcol85-Gal4/+; UAS-PtenRNAi/+) (1.4% (n = 51)) (F). (G-H) The number of lamellocytes in the circulation of col>hdcRNAi larvae is reduced when simultaneously Akt (Pcol85-Gal4,UAS-hdcRNAi/+; UAS-AktRNAi/+) (0.08% (n = 41)) (G) or raptor (Pcol85-Gal4,UAS-hdcRNAi/+; UAS-raptorRNAi/+) (0.2% (n = 34)) is silenced (H). (I) Overexpression of hdc reduces lamellocyte numbers in the circulation of col>PtenRNAi larvae (Pcol85-Gal4/+; UAS-PtenRNAi/UAS-hdc.S) (0.14% (n = 36)). n refers to the number of larvae analyzed. Nuclei are visualized by DAPI (blue). Scale bar:20 μm. (J) A scatter dot plot showing the percentage of lamellocytes in the circulation of larvae from the genotypes presented in panels (A-I). Each dot in the graph represents a single larva. Data were analyzed using ANOVA with Tukey’s test for multiple comparisons, * p ≤ 0.05, ** p ≤ 0.01, *** p ≤ 0.001, **** p ≤ 0.0001. (TIF) [file pgen.1011448.s001.tif]

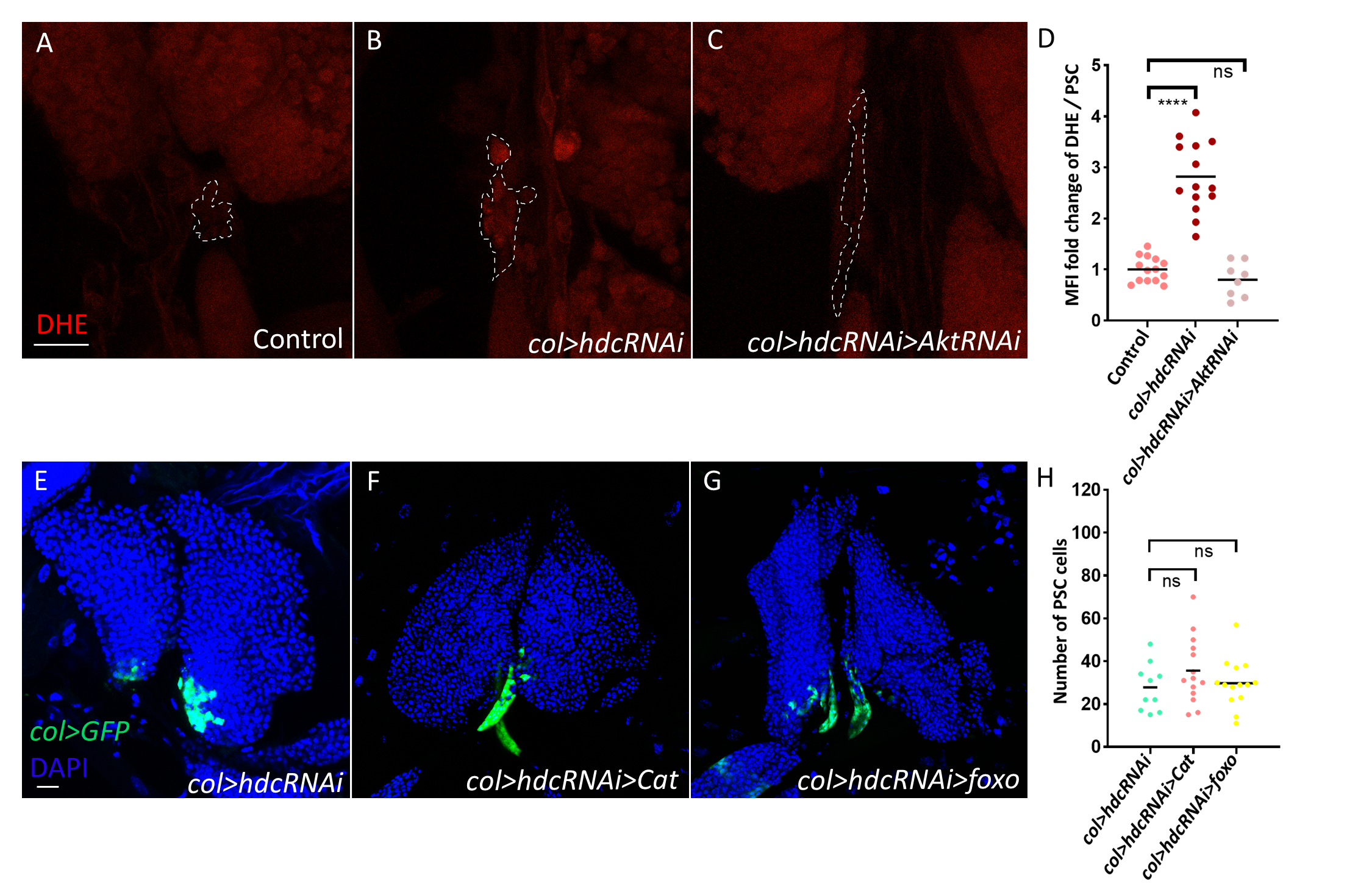

Supplement: S2 Fig — (A-C) Silencing hdc results in higher ROS levels in the PSC (col>GFP positive area) as shown by the oxidation of the ROS indicator DHE dye (red) (Pcol85-Gal4,UAS-2xEGFP/UAS-hdcRNAi) (n = 14) (B), which is not observed in the control (Pcol85-Gal4,UAS-2xEGFP/+) (n = 14) (A) or when Akt is silenced simultaneously with hdc in the PSC (Pcol85-Gal4,UAS-hdcRNAi/+; UAS-AktRNAi/UAS-2xEGFP) (n = 8) (C). n refers to the number of lymph gland lobes analyzed. Scale bar: 20 μm. (D) A scatter dot plot showing the mean fluorescence intensity (MFI) of DHE (represented in fold change in comparison to the control) from the genotypes presented in the panels (A-C). Each dot in the graph represents a PSC from one lobe. Data were analyzed using ANOVA with Tukey’s test for multiple comparisons, **** p ≤ 0.0001, ns: non-significant. (E-G) Overexpressing Cat (Pcol85-Gal4,UAS-hdcRNAi/UAS-Cat; UAS-2xEGFP/+) (average number of PSC cells = 35, n = 14) (F) or foxo (Pcol85-Gal4,UAS-hdcRNAi/UAS-foxo; UAS-2xEGFP/+) (average number of PSC cells = 29, n = 14) (G) did not change the size of the PSC (number of col>GFP positive cells) of col>hdcRNAi (Pcol85-Gal4,UAS-hdcRNAi/+; UAS-2xEGFP/+) (average number of PSC cells = 27, n = 10) larvae (E) (blue: nuclei, green: PSC). n refers to the number of lymph gland lobes analyzed. Scale bar: 20 μm. (H) A scatter dot plot showing PSC cell number in larvae from the genotypes presented in panels (E-G). Each dot in the graph represents a PSC from one lymph gland lobe. Data were analyzed using ANOVA with Tukey’s test for multiple comparisons, ns: non-significant. (TIF) [file pgen.1011448.s002.tif]

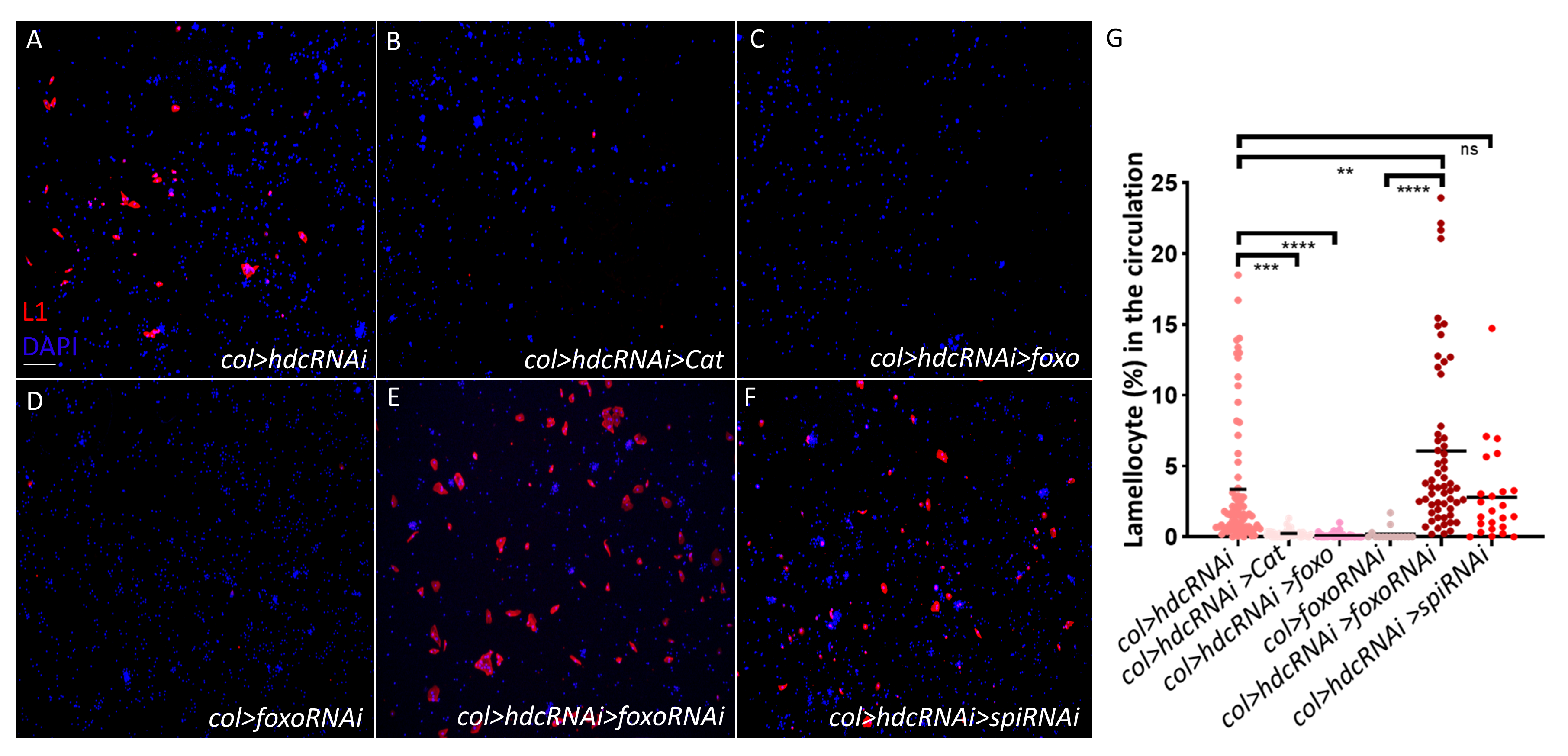

Supplement: S3 Fig — (A-C) Overexpression of Cat (Pcol85-Gal4,UAS-hdcRNAi/UAS-Cat) (0.25% (n = 40)) (B) or foxo (Pcol85-Gal4,UAS-hdcRNAi/UAS-foxo) (0.08% (n = 51)) (C) rescues lamellocyte differentiation in hdc silenced larvae (Pcol85-Gal4,UAS-hdcRNAi/+) (3.3% (n = 77)) (A). (D-E) Silencing foxo simultaneously with hdc (Pcol85-Gal4,UAS-hdcRNAi/+; UAS-foxoRNAi/+) enhances the lamellocyte differentiation phenotype associated with hdc silencing (6% (n = 58)) (E), while silencing foxo alone in the niche does not lead to lamellocyte differentiation (Pcol85-Gal4/+; UAS-foxoRNAi/+) (0.19% (n = 15)) (D). (F) Silencing spi in the niche does not affect lamellocyte numbers in the circulation of col>hdcRNAi larvae (Pcol85-Gal4,UAS-hdcRNAi/UAS-spiRNAi) (2.8% (n = 24)) (blue: nuclei, red: lamellocytes). n refers to the number of larvae analyzed. Scale bar: 20 μm. (E) A scatter dot plot quantifying lamellocyte numbers in larvae from the genotypes presented in panels (A-F). Each dot in the graph represents a single larva. Data were analyzed using ANOVA with Tukey’s test for multiple comparisons, ** p ≤ 0.01, *** p ≤ 0.001, **** p ≤ 0.0001, ns: non-significant. (TIF) [file pgen.1011448.s003.tif]

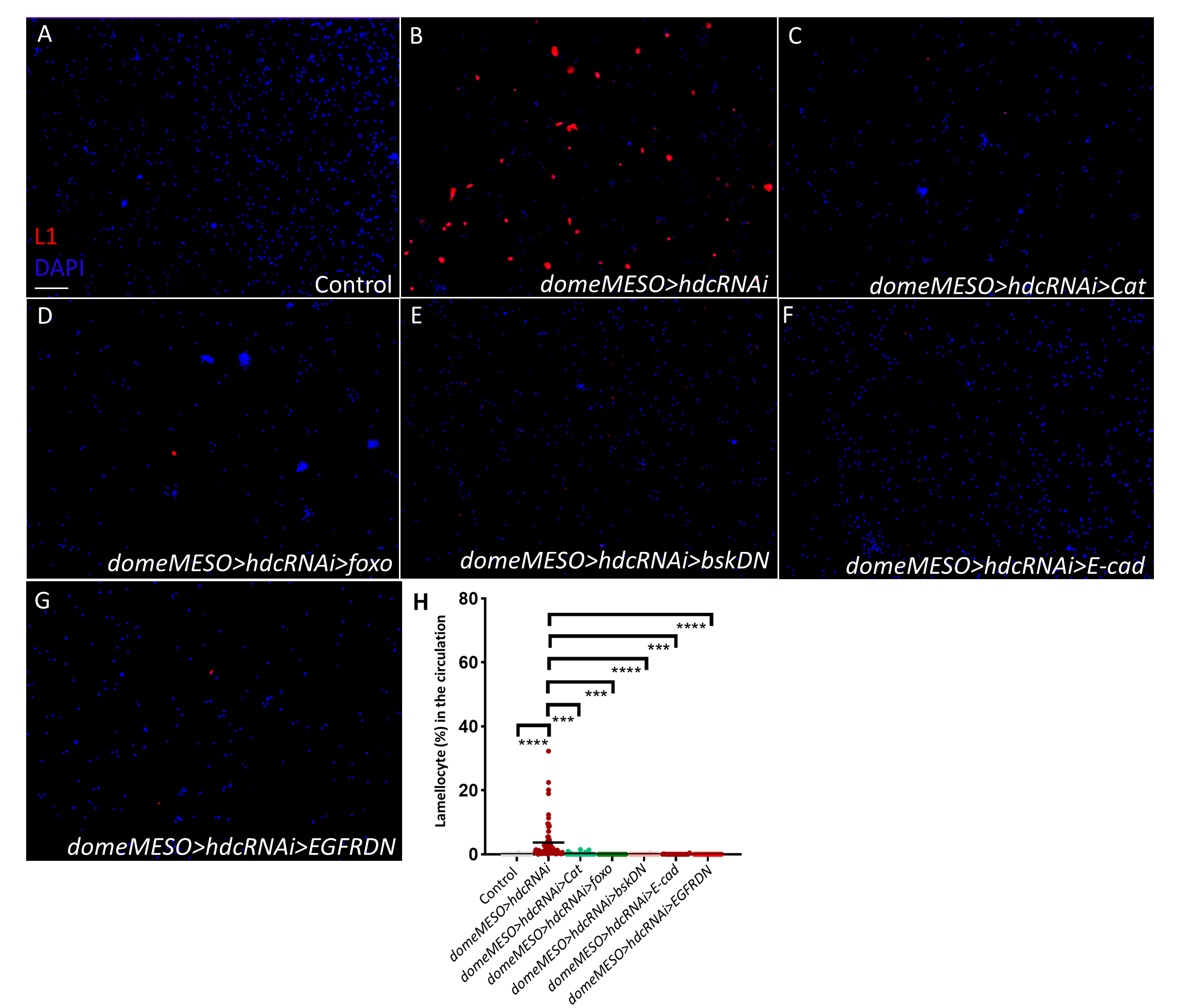

Supplement: S4 Fig — (A-B) Silencing hdc in the MZ leads to the appearance of lamellocytes in the circulation (UAS-hdcRNAi/+; domeMESO-GAL4,UAS-2xEGFP/+) (3.7% (n = 67)) (B), which are normally not present in the control (domeMESO-GAL4,UAS-2xEGFP/+) (0.04% (n = 33)) (A). (C-G) Overexpression of Cat (UAS-hdcRNAi/UAS-Cat; domeMESO-GAL4,UAS-2xEGFP/+) (0.2% (n = 24)) (C) or foxo (UAS-hdcRNAi/UAS-foxo; domeMESO-GAL4,UAS-2xEGFP/+) (0% (n = 22)) (D) or a dominant negative form of bsk (UAS-hdcRNAi/+; domeMESO-GAL4,UAS-2xEGFP/UAS-bsk53R) (0.01% (n = 26)) (E), E-cad (UAS-hdcRNAi/+; domeMESO-GAL4,UAS-2xEGFP/UAS-E-cad) (0.03% (n = 19)) (F) or a dominant negative form of EGFR (UAS-hdcRNAi/UAS-EGFR.DN; domeMESO-GAL4,UAS-2xEGFP/UAS-EGFR.DN) (0% (n = 26)) (G) is able to rescue hdc lamellocyte phenotype (blue: nuclei, red: lamellocytes). n refers to the number of larvae analyzed. Scale bar: 20 μm. (H) A scatter dot plot showing percentage of lamellocytes in the circulation of larvae from the genotypes presented in panels (A-G). Each dot in the graph represents one single larva. Data were analyzed using ANOVA with Tukey’s test for multiple comparisons, *** p ≤ 0.001, **** p ≤ 0.0001. (TIF) [file pgen.1011448.s004.tif]

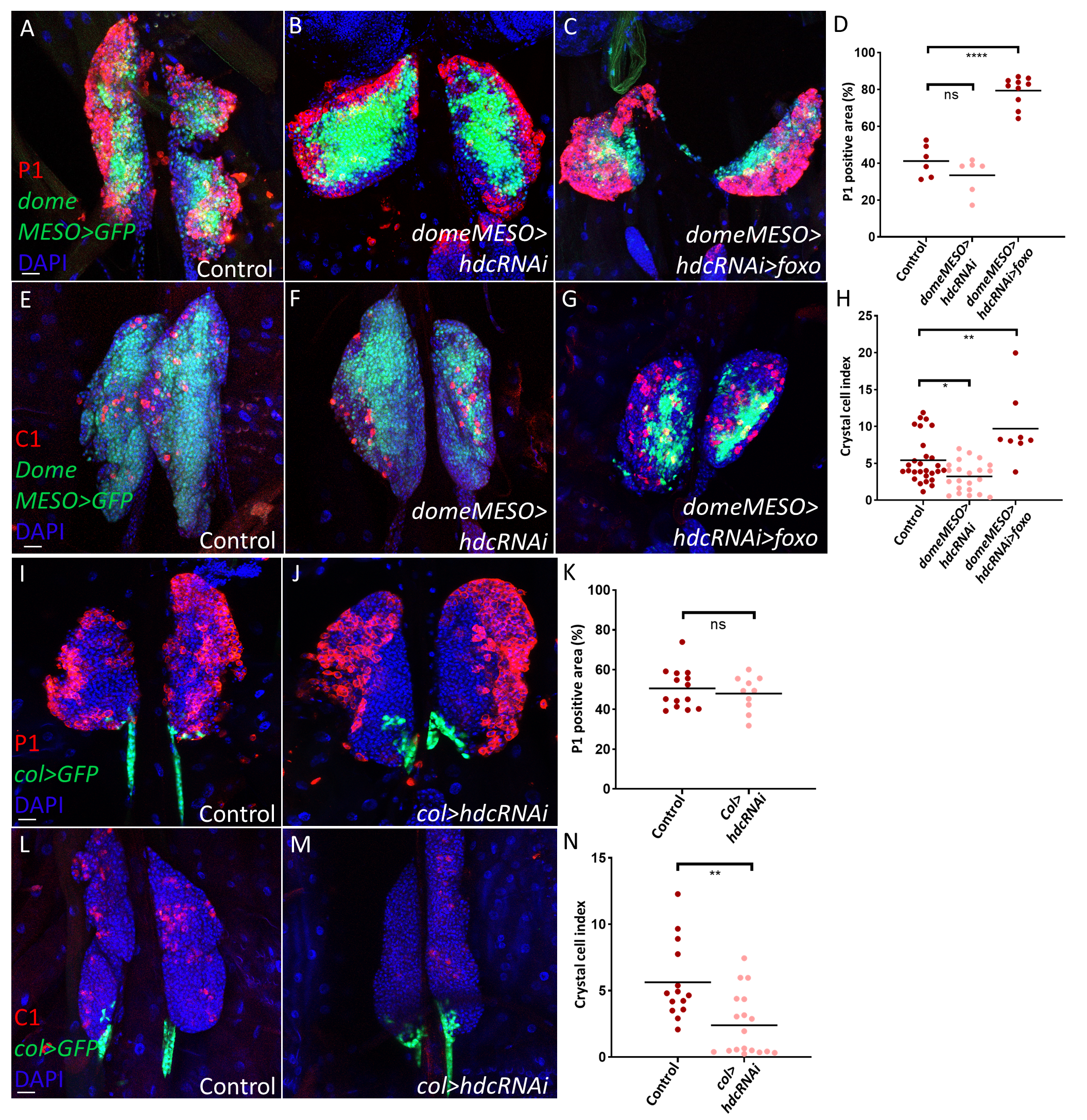

Supplement: S5 Fig — (A-C) Silencing hdc in the MZ (UAS-hdcRNAi/+; domeMESO-GAL4,UAS-2xEGFP/+) does not affect P1 positive (plasmatocyte) area percentage per anterior lobe (average = 33%, number of lobes = 6) (B), while overexpressing foxo in dome>hdcRNAi larvae (UAS-hdcRNAi/UAS-foxo; domeMESO-GAL4,UAS-2xEGFP/+) significantly increases it (average = 79%, number of lobes = 10) (C) in comparison to the control (domeMESO-GAL4,UAS-2xEGFP/+) (average = 41%, number of lobes = 6) (A) (blue: nuclei, green: MZ, red: plasmatocytes). Scale bar: 20 μm. (D) A scatter dot plot showing P1 positive (plasmatocyte) area percentage per anterior lobe from the genotypes in the panels (A-C). Each dot in the graph represents one anterior lobe. Data were analyzed using ANOVA with Tukey’s test for multiple comparisons, **** p ≤ 0.0001, ns: non-significant. (E-G) Silencing hdc in the MZ (UAS-hdcRNAi/+; domeMESO-GAL4,UAS-2xEGFP/+) significantly reduces the crystal cell index (average = 3.2, number of lobes = 22) (F), while overexpressing foxo in dome>hdcRNAi larvae (UAS-hdcRNAi/UAS-foxo; domeMESO-GAL4,UAS-2xEGFP/+) significantly increases it (average = 9.7, number of lobes = 8) (G) in comparison to the control (domeMESO-GAL4,UAS-2xEGFP/+) (average = 5.4, number of lobes = 28) (E) (blue: nuclei, green: MZ, red: crystal cells). Scale bar: 20 μm. (H) A scatter dot plot quantifying crystal cell index from the genotypes in the panels (E-G). Each dot in the graph represents one anterior lobe. Data were analyzed using ANOVA with Tukey’s test for multiple comparisons, * p ≤ 0.05, ** p ≤ 0.01. (I-J) Silencing hdc in the PSC (UAS-hdcRNAi/+; domeMESO-GAL4,UAS-2xEGFP/+) does not affect P1 positive (plasmatocyte) area percentage per anterior lobe (Pcol85-Gal4,UAS-2xEGFP/UAS-hdcRNAi) (average = 47%, number of lobes = 10) (J), in comparison to the control (Pcol85-Gal4,UAS-2xEGFP/+) (average = 50%, number of lobes = 14) (I) (blue: nuclei, green: PSC, red: plasmatocytes). Scale bar: 20 μm. (K) A scatter dot plot showing P1 positi [file pgen.1011448.s005.tif]

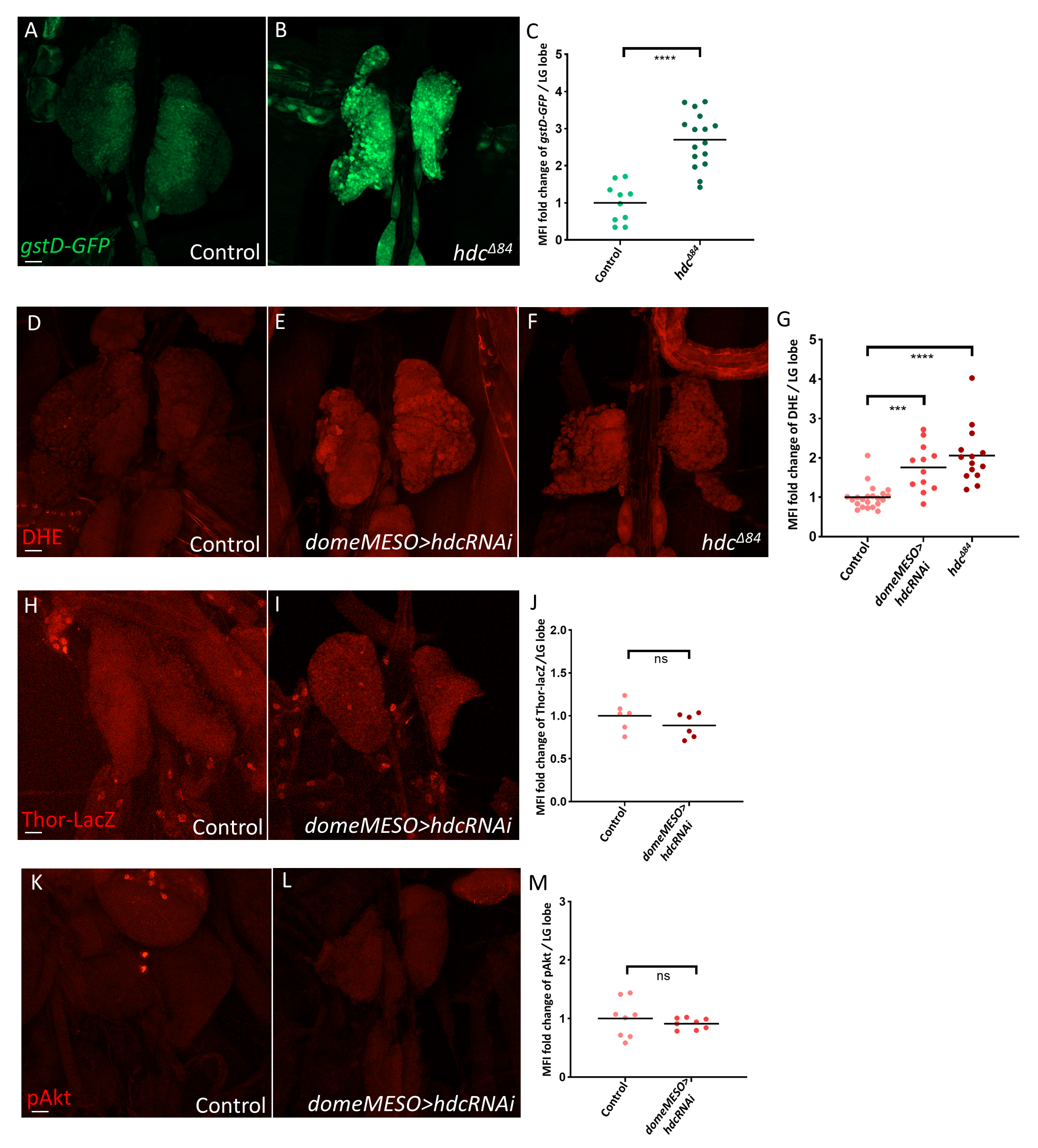

Supplement: S6 Fig — (A-B) hdcΔ84 null mutants (gstD-GFP; hdcΔ84) show higher gstD-GFP induction (n = 16) (B) in comparison to the control (gstD-GFP) (n = 10) (A) (green: gstD-GFP). n refers to the number of lymph gland lobes analyzed. Scale bar: 20 μm. (C) A scatter dot plot showing the fold change increase (average = 2.7 folds) in the MFI of gstD-GFP in the anterior lymph gland lobes of hdcΔ84 null larvae (gstD-GFP; hdcΔ84) in comparison to the control (gstD-GFP). Each dot in the graph represents one anterior lobe. Data were analyzed using two-tailed unpaired Student’s t-test, **** p ≤ 0.0001. (D-F) domeMESO>hdcRNAi larvae (n = 12) (UAS-hdcRNAi/+; domeMESO-Gal4/+) (E) and hdcΔ84 null mutants (n = 14) (hdcΔ84) (F) show higher DHE florescence in comparison to the control (w118) (n = 22) (D) (red: DHE). n refers to the number of lymph gland lobes analyzed. Scale bar: 20 μm. (G) A scatter dot plot showing the fold change increase in the MFI of DHE in the anterior lymph gland lobes of domeMESO>hdcRNAi (average = 1.7 folds) and hdcΔ84 null larvae (average = 2 folds) in comparison to the control. Each dot in the graph represents one anterior lobe. Data were analyzed using ANOVA with Tukey’s test for multiple comparisons, *** p ≤ 0.001, **** p ≤ 0.0001. (H-I) Silencing hdc does not affect the transcription of Thor as detected by an anti-lacZ staining for the Thor-lacZ reporter (UAS-hdcRNAi/+; domeMESO-Gal4,UAS-2xEGFP/Thor-lacZ) (n = 6) (I) in comparison to the control (domeMESO-Gal4,UAS-2xEGFP/Thor-lacZ) (n = 6) (H) (red: Thor-LacZ). Scale bar: 20 μm. (J) A scatter dot plot showing fold change in MFI of Thor-LacZ per anterior lobe of (UAS-hdcRNAi/+; domeMESO-Gal4/Thor-lacZ) larvae (average = 0.8) compared to the control (domeMESO-Gal4/Thor-lacZ) (average = 1). Each dot in the graph represents one anterior lobe. Data were analyzed using two-tailed unpaired Student’s t-test, ns: non-significant. (K-L) Silencing hdc does not affect the levels of pAkt in the lymph gland (UAS-hdcRNAi/+; domeMESO-G [file pgen.1011448.s006.tif]

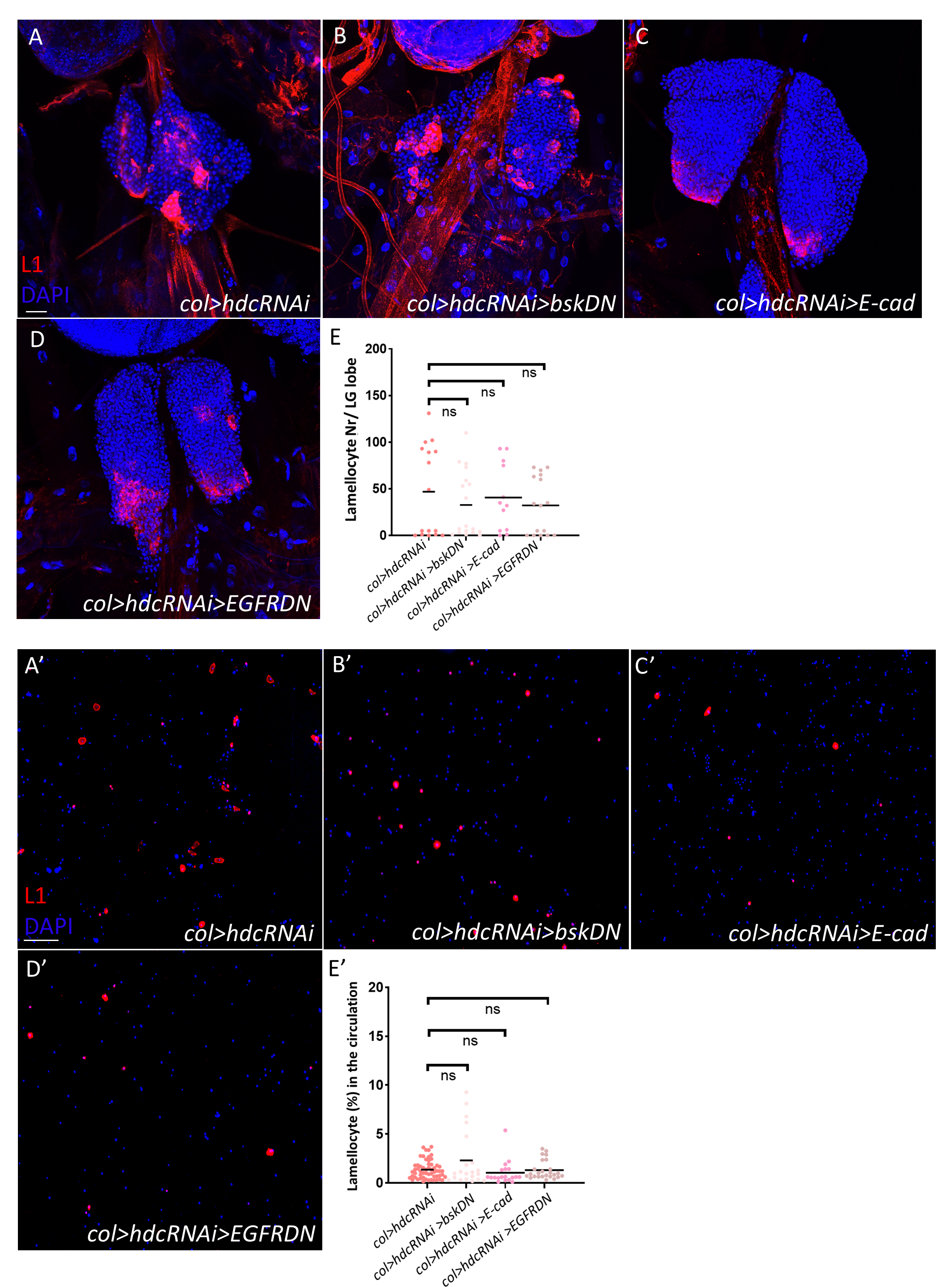

Supplement: S7 Fig — (A-D) Expressing a dominant negative version of bsk (Pcol85-Gal4,UAS-hdcRNAi/+; UAS-bsk53R/+) (n = 18) (B), or overexpressing E-cad (Pcol85-Gal4,UAS-hdcRNAi/+; UAS-E-cad/+) (n = 12) (C) or a dominant negative version of EGFR (Pcol85-Gal4,UAS-hdcRNAi/UAS-EGFR.DN; UAS-EGFR.DN/+) (n = 16) (D) or does not affect lamellocyte differentiation in the lymph glands of col>hdcRNAi larvae (Pcol85-Gal4,UAS-hdcRNAi/+) (n = 16) (A) (blue: nuclei, red: lamellocytes). n refers to the number of lymph gland lobes analyzed. Scale bar: 20 μm. (E) The number of lamellocytes per lymph gland lobe in the genotypes presented in panels (A-D). Each dot in the graph represents one lymph gland lobe. Data were analyzed using ANOVA with Tukey’s test for multiple comparisons, ns: non-significant. (A’-D’) Expressing a dominant negative version of bsk (Pcol85-Gal4,UAS-hdcRNAi/+; UAS-bsk53R/+) (2.3% (n = 21)) (B’), or overexpressing E-cad (Pcol85-Gal4,UAS-hdcRNAi/+; UAS-E-cad/+) (1% (n = 19)) (C’) or a dominant negative version of EGFR (Pcol85-Gal4,UAS-hdcRNAi/UAS-EGFR.DN; UAS-EGFR.DN/+) (1.3% (n = 25)) (D’) does not affect lamellocyte differentiation in the circulation of col>hdcRNAi larvae (Pcol85-Gal4,UAS-hdcRNAi/+) (1.3% (n = 57)) (A’). (blue: nuclei, red: lamellocytes). n refers to the number of larvae analyzed. Scale bar: 20 μm. (E’) A scatter dot plot quantifying lamellocyte numbers in larvae from the genotypes presented in panels (A’-D’). Each dot in the graph represents a single larva. Data were analyzed using ANOVA with Tukey’s test for multiple comparisons, ns: non-significant. (TIF) [file pgen.1011448.s007.tif]

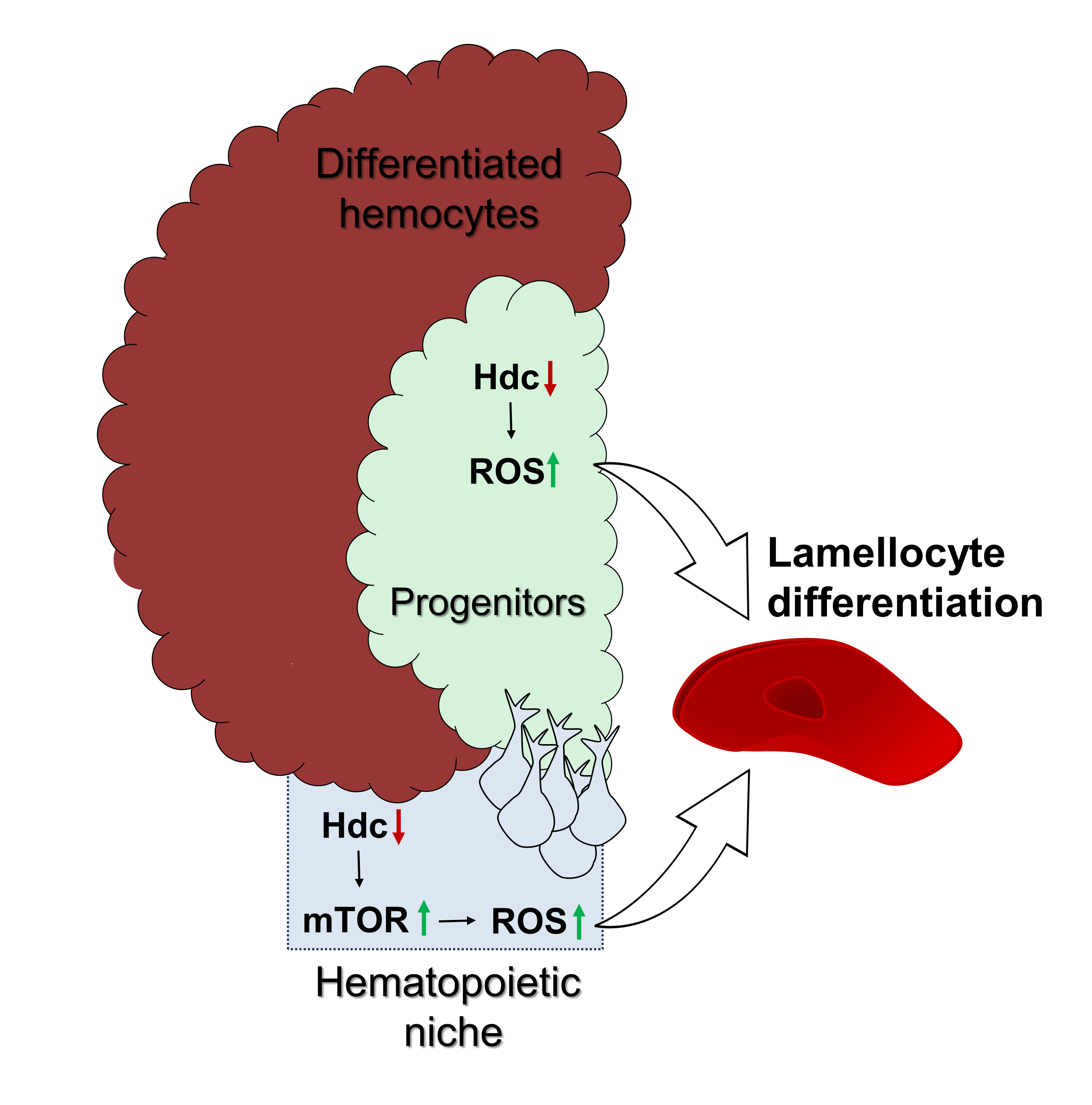

Supplement: S1 File — (TIF) [file pgen.1011448.s009.tif]
